# Fractal dendrite-based electrically conductive composites for laser-scribed flexible circuits

**DOI:** 10.1038/ncomms9150

**Published:** 2015-09-03

**Authors:** Cheng Yang, Xiaoya Cui, Zhexu Zhang, Sum Wai Chiang, Wei Lin, Huan Duan, Jia Li, Feiyu Kang, Ching-Ping Wong

**Affiliations:** 1 Division of Energy and Environment, Graduate School at Shenzhen, Tsinghua University, Shenzhen 518055, China; 2 School of Materials Science and Engineering, Georgia Institute of Technology, 771, Ferst Dr, Atlanta, Georgia 30332, USA; 3 School of Materials Science and Engineering, Tsinghua University, Beijing 100084, China; 4 Department of Electronic Engineering, The Chinese University of Hong Kong, Shatin, Hong Kong, China; 6 Present address: Apple Inc, 19333 Vallco Pkwy, Cupertino, California 95014, USA

## Abstract

Fractal metallic dendrites have been drawing more attentions recently, yet they have rarely been explored in electronic printing or packaging applications because of the great challenges in large-scale synthesis and limited understanding in such applications. Here we demonstrate a controllable synthesis of fractal Ag micro-dendrites at the hundred-gram scale. When used as the fillers for isotropically electrically conductive composites (ECCs), the unique three-dimensional fractal geometrical configuration and low-temperature sintering characteristic render the Ag micro dendrites with an ultra-low electrical percolation threshold of 0.97 vol% (8 wt%). The ultra-low percolation threshold and self-limited fusing ability may address some critical challenges in current interconnect technology for microelectronics. For example, only half of the laser-scribe energy is needed to pattern fine circuit lines printed using the present ECCs, showing great potential for wiring ultrathin circuits for high performance flexible electronics.

## Abstract (web summary)

Electrically conductive composites are increasingly being studied for their favourable mechanical and electrical properties. Here, the authors employ a new synthetic strategy to attain fractal silver micro-dendrites which can be laser-scribed for implementation in flexible printed microelectronics.

## Graphical abstract

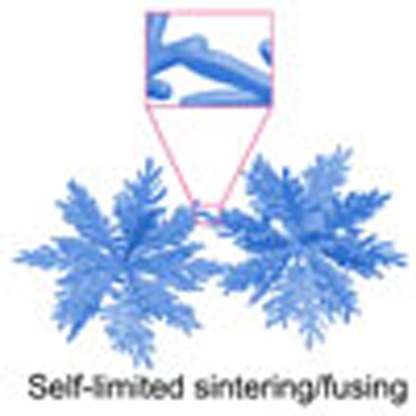

Future electronic devices with smaller and thinner form factors, coupled with green initiatives and cost reduction efforts, have boosted the research and development of novel micro-/nano-metallic printing materials that possess unique advantages in advanced interconnect technology^1^,^2^,^3^,^4^. Metallic dendrites with beautiful and intriguing three-dimensional (3D) micro-/nanostructures are such a motif, along with their great potentials in various other areas^5^,^6^,^7^,^8^,^9^. Despite tremendous efforts on their syntheses, it is still challenging to tailor metallic dendrites precisely in a scalable manner. Until now, a few synthetic methods of metallic dendrite particles have been reported, including electrochemical method^10^,^11^,^12^, sono-electrochemical method^13^, hydrothermal method^14^, ultrasonic wave-assisted method^15^, controlled seeding method^9^,^16^,^17^,^18^, zinc plate replacement reaction^19^, galvanic replacement method^20^,^21^ and so forth. Even though the above methods are capable to prepare metallic dendrites effectively, it is still very difficult to precisely tailor their hierarchical structures effectively, and thus hinders the fundamental understanding of their percolation properties in composites and further evaluation of their engineering value. As a consequence, applications of 3D metallic dendrites in electronics remain a virgin land. The present paper elucidates a scalable preparation of 3D dendritic silver. When being used as fillers for electrically conductive composites (ECCs), the dendritic silver filler particles reveal an unprecedentedly low percolation threshold in a 3D space, promising a breakthrough in flexible printed electronics.

ECCs have found extensive applications in die attachments, solar cell panels, radio frequency identification antennas, flexible printed circuits, electromagnetic shielding and many other technologies^1^. One most important measure of the electrical property of ECCs is the spatial percolative efficiency, which is related to the species, size (and size distribution), geometry (including shape and aspect ratio), dispersion and distribution of the conductive fillers in a polymer matrix^22^. Although one-dimensional (tubes, wires) and two-dimensional (2D) fillers (platelets and discs) have the intrinsic advantage over 3D (particle) fillers in percolation threshold^23^, some disadvantages and limitations have emerged for one-dimensional fillers and 2D fillers in the applications of ECCs. For example, the shear force during printing and dispensing processes may induce alignment of the fillers, causing anisotropicity of the composite with weakened conductivity normal to the filler-aligned direction(s). This results in a high leakage (even shorting) along the alignment direction(s) and a high resistance in the normal direction—usually the interconnect direction, although it is disadvantageous for some applications such as anisotropically conductive adhesive/films. Other than turning the knob on filler geometry, enormous efforts have been made to push the percolation efficiency beyond the intrinsic limit determined by the geometry of a specific filler material in a regular case of random dispersion and distribution. For example, mixing fillers of different sizes and shape can be a means of adjusting the aggregation state of the fillers in the ECCs^2^,^24^; the filler surface can be modified to render a better Ohmic contact between adjacent fillers^25^,^26^,^27^. However, some of the methods may add certain complexities to industry-scale formulation/preparation of ECCs that desires a large process window in mixing, easy-to-reach homogeneity, and long shelf life and so on. In these regards, simple mixing of 3D metallic fillers with a polymer matrix is beneficial for process simplification, quality control, and cost reduction for ECC applications. Yet the fundamental challenge is how to break the intrinsic percolation limit of 3D fillers that are randomly dispersed and distributed in a matrix. Towards that end, we demonstrate that micro-structuring of the surfaces of the 3D fillers provides a new path.

In the present study, we introduce silver-based 3D fractal dendrites (FDs) into engineering polymer resins, and have achieved an extremely low percolation threshold of 0.97 vol% (8 wt%) of silver. This is attributed to the unique nano-sized rims of the metallic dendrites, which fuse with each other through a simple sintering process at low temperature, forming a conductive network at ultra-low filler loading, which can be attributable to the low-temperature sintering ability of the nano-sized silver structures^28^,^29^. The simulated percolation threshold using Monte Carlo computation shows good agreement with the experimental results, from both the perspectives of spatial spanning and contact probability (see Supplementary Information).

## Results

### Laser-scribe patterning applications

Besides the ultra-low percolation threshold, the FDs also show some important advantages in the laser-scribe circuit patterning process. When heated by a laser, the outer rims of the FDs melt. As a result, the scribed area of the ECC shrinks, resulting in non-conductive regions along the otherwise conductive paths. As compared with some of the previous works about light (flash)-triggered sinter-interconnecting for some nanomaterials, for example, graphene, polyaniline and metal^4^,^30^,^31^, the current phenomenon is called fusing cut-off, an essential property of the FD-based ECCs (FD-ECCs) for laser-scribe patterning of peripheral circuits on thin plastic films for flexible touch panels. As demonstrated here, patterning 20 μm lines of the FD-ECC requires only half of the laser power that is required to pattern a commercial benchmark ECC (FTL-630LE, FP Co Ltd, Korea). Taking the properties of ultra-low percolation threshold and low-temperature fusing cut-off, we consider the FD-ECCs a prototype of the essential interconnect materials for next-generation mobile/flexible electronics.

### Controllable preparation of FDs

**Figure 1:**
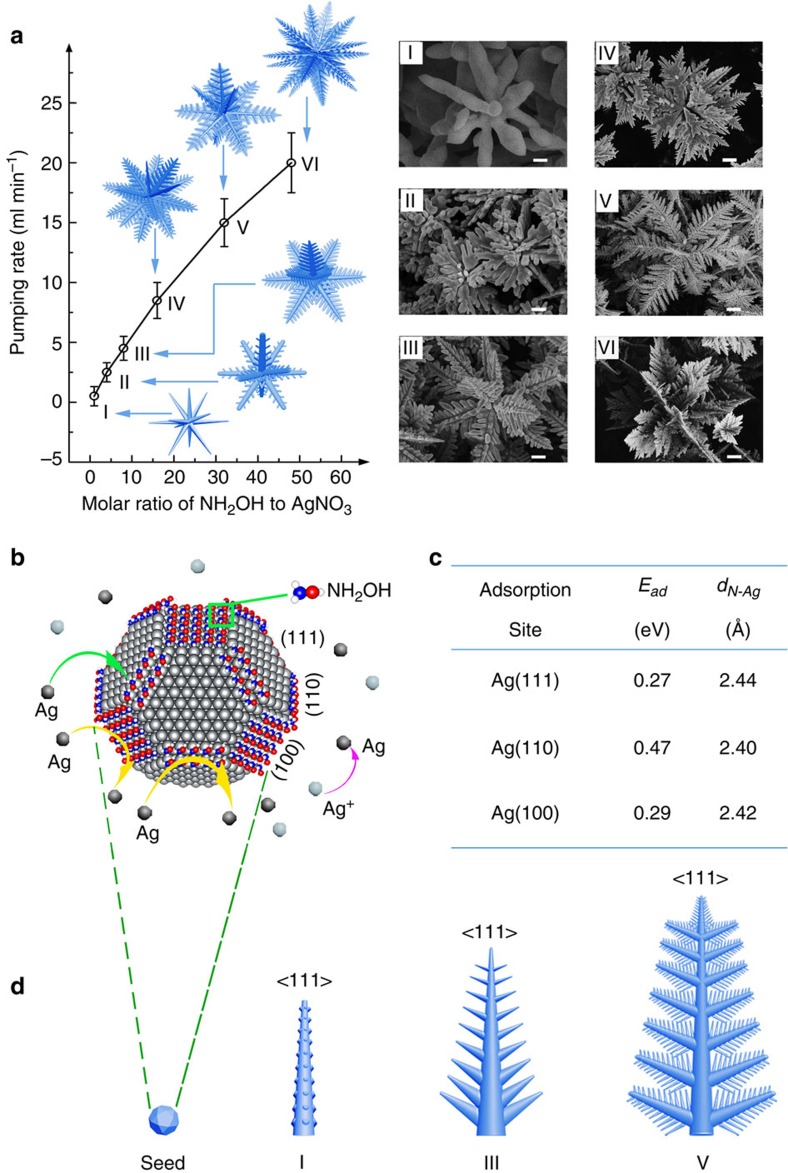
Schematic representations of the preparation window and growth mechanism of the FDs. (**a**) (left) Schematic representations of the FDs grown at various molar ratios and pumping rates (other form factors were the same). (right) The corresponding SEM images of the FDs with six typical morphologies: (I) FD I, size 1.5–2.5 μm; (II) FD II, size 1.8–3 μm; (III) FD III, size 3–5.5 μm; (IV) FD IV, size 3.5–6.5 μm; (V) FD V, size 5–7 μm; and (VI) FD VI, size 5.5–7.5 μm. (Insets I–IV scale bars are 200, 300, 400 and 600 nm, respectively. Insets V and VI scale bars are 1μm. (**b**) A schematic representation showing the adsorption of NH_2_OH on silver surfaces and the plausible formation process of FDs. (**c**) A table listing the adsorption energy (*E*_ad_) and N-Ag distance (*d*_N-Ag_) of NH_2_OH adsorption on (4 × 4) silver surfaces. The N-Ag distance is defined as the distance between the N atom in NH_2_OH and its underlying Ag atom. (**d**) The detailed morphology of a virtual branch of various FDs (I, III and VI) as shown in (**a**).

As shown in Fig. 1a, by adjusting the feeding rate and molar ratio in the synthesis, we are able to obtain FDs with various fractal structures and sizes through a multi-channel micro-droplet reaction (see Supplementary Fig. 1). It is found that hydroxylamine plays two key roles in the reactions as the reducing agent and the surface coupling agent. With the increase of the concentration of hydroxylamine, the excess hydroxylamine can accelerate the reaction rate, resulting in more nucleation sites and a higher growing speed. For example, when the concentration of hydroxylamine was 0.06 M, with the feeding rate of 0–5 ml min^−1^, urchin-like FDs are obtained (type I, size ∼2 μm, D90: 1.2–2.7 μm, rod-like branches). When the concentration of hydroxylamine is increased to 0.24 M, with the feeding rate of 4–10 ml min^−1^, FDs with a secondary fractal structure are obtained (for example, type III, size ∼5 μm, D90: 3.2–5.6 μm). Further increasing the concentration of hydroxylamine to 0.48 M (feeding rate of 10–20 ml min^−1^) generates FDs with secondary and tertiary structures (for example, type VI, size ∼ 6.5 μm, D90: 6.0–8.0 μm). Details on experimental and categorization are summarized in Supplementary Fig. 2 and Supplementary Table 1. Using this reaction system, we can prepare 3D Au FDs as well, as shown in Supplementary Fig. 4.

### Study of the FDs growth mechanism

The growth mechanism of the FDs can be better understood from the first-principles quantum-mechanical calculations. Density functional theory modelling results show that the binding energy of (110), (100) and (111) planes are 0.47, 0.29 and 0.27 eV, respectively, and when the concentration of NH_2_OH increases, the coverage of NH_2_OH on the silver planes increases relatively (from 0.06 to 0.25), the binding energy of (110), (100) and (111) planes changes to 0.51, 0.41 and 0.34 eV, as shown in Fig. 1c, Supplementary Fig. 5 to Supplementary Fig. 8 and Supplementary Tables 2–4. The calculation results suggest that NH_2_OH molecules (with optimized structure) tend to bind the (110) and (100) planes of the silver nanocrystals and lead to passivation of the (110) and (100) planes but promote the selective deposition of Ag atoms along the (111) planes. It is noted that the increase of the concentration of hydroxylamine may not only improve the facet adsorption selectivity but also accelerate the reduction of Ag^+^ to Ag^0^, which renders faster Ag atom deposition on the seed crystals. Kinetically, when the latter overwhelms former, deviation from the selective growth rule can take place, leading to the formation of new (111) facets, as shown in Fig. 1b,d. This competing process may lead to more complex fractal structures, such as the tertiary branches. Further study is still undergoing.

### Characterizations of the FDs structural and optical features

**Figure 2:**
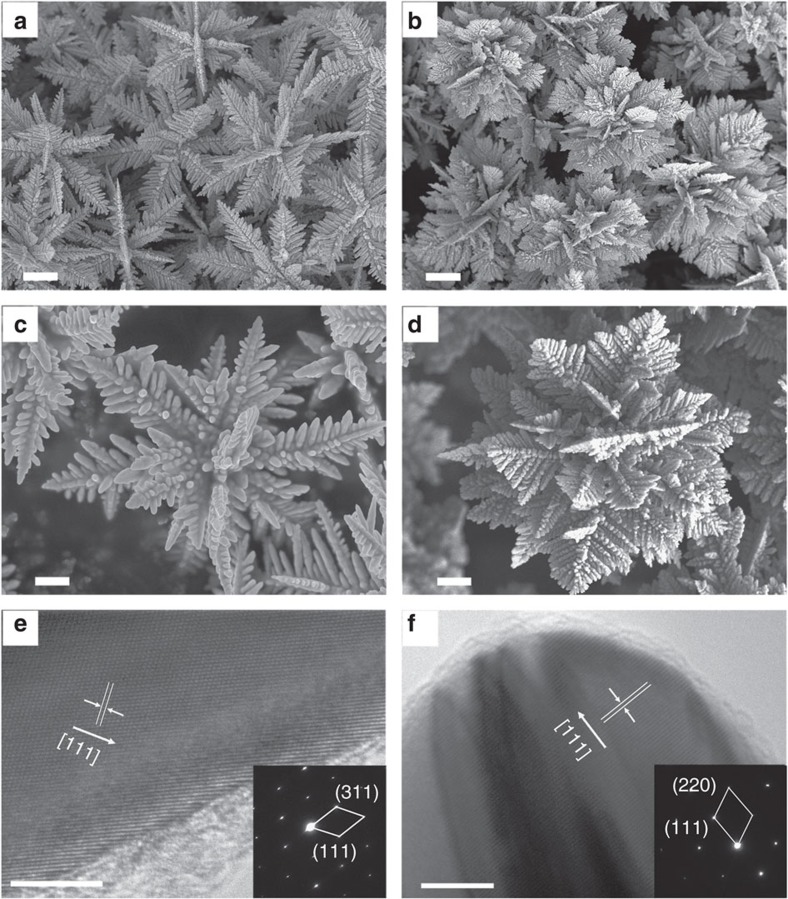
SEM and TEM images of the FDs. (**a**) Low-magnification SEM image of FD III; (**b**) low-magnification SEM image of FD VI (scale bars, 1 μm (**a**,**b**)); (**c**) high-magnification SEM image of FD III; (**d**) high-magnification SEM image of FD VI (scale bars, 500 nm (c,d)); (**e**) HRTEM image of the rim of FD III; (**f**) HRTEM images of the rims of FD VI (scale bars,5 nm (**e**,**f**)) (insets: the corresponding selected area electron diffraction patterns and indexing of the nanotip region.)

For the characterizations of the FDs, we focus on types III and VI as the most representative FDs with secondary and tertiary structure, respectively, because theirs sizes are around 5 μm—the size generally used in ECC applications for microelectronics. The scanning electron microscopy (SEM) images of FD III and FD VI are shown in Fig. 2a-d, and the high-resolution SEM images (Fig. 2c,d) show that FD III have two levels of branches with both micro-size and nano-size features, while FD VI has a delicate tertiary fractal structure with more abundant nanotips. The crystalline features of the FDs were examined by transmission electron microscopy (TEM) and X-ray diffraction, as shown in Fig. 2e,f, Supplementary Figs 9 and 10. Both the selected area electron diffraction patterns and lattice images of these two FDs suggest that the branches of the FDs be monocrystalline, with the silver atoms collected on the close-packed lattice of (111) facet of the root crystal. The secondary structure and the tertiary structure of each FD particle are mirror-symmetrical individually. FDs can act as good substrate for surface-enhanced Raman scattering applications as shown in Supplementary Fig. 11, and their specific surface areas are shown in Supplementary Fig. 12. The monocrystalline microstructure along each branch ensures excellent electrical conductivity compared with otherwise polycrystalline microstructure in which grain boundary scattering contributes importantly to the resistivity.

### Investigation of the sintering behaviour of FDs

**Figure 3:**
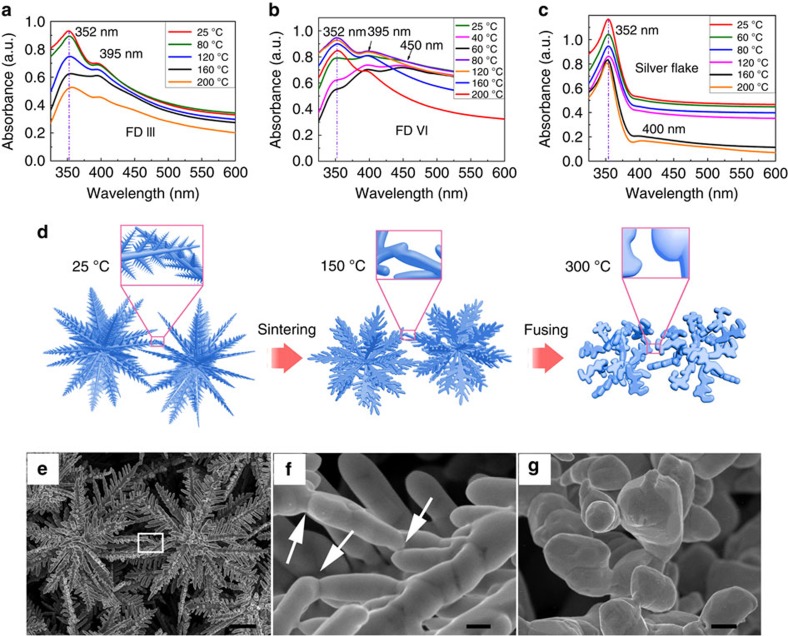
Morphologies and the optical absorption properties of the FDs. (**a**–**c**): UV/vis absorption spectra (reflective mode) of the dry powder samples. (**a**) FD III, (**b**) FD VI and (**c**) silver flake (control sample) after annealing at different temperatures in argon atmosphere; (**d**) schematic representation showing the sintering behaviours of FD VI at different temperatures; (**e**) an SEM image of two adjacent FD VI, after sintering at 150 °C; (**f**) a magnified image of the selected area in (**e**), with the arrows pointing to the fused tips of the adjacent FD VI; (**g**) an SEM image of FD VI after annealed at 300 °C in argon (scale bars, 1 μm, 200 nm and 200 nm, respectively (**e**–**g**)).

In a 3D space, the FDs tend to aggregate with a minimized energy state. With adequate thermal energy, sintering of the nano-sized rims starts to take place. Differential scanning calorimetry analysis suggests that with the increase of temperature (from 25 to 300 °C), the endothermic process is accelerated, confirming a comprehensive sintering process of the FD particles (see Supplementary Fig. 18); thermal gravimetric analysis suggests that the weight loss is <0.6% when heated to 300 °C, which proves a clean surface of FDs. More detailed sintering process can be monitored via observing the evolution of the optical absorption characteristic which is related to the surface plasmon resonance (SPR) property. Since the SPR is the resonant oscillation of conduction electrons at the metal–dielectric interfaces stimulated by incident light, when the incident photons (generated during the UV–vis measurement) match the natural frequency of surface electrons oscillating, a scattering signal can be probed. More importantly, the scattering signal is closely related to the size, shape, and dielectric environment of the metal sample. Thus the UV–vis measurement results can excellently reflect the morphology of the individual FD and their association status change. The optical absorption property of the FDs at various thermal sintering stages can be analyzed by UV/vis spectroscopy. The light absorption characteristics of both the FDs and the 2D silver flakes (control sample) after a stepwise heating process are shown in Fig. 3a–c and the corresponding sample heating curve is shown in Supplementary Fig. 13. It is found that FD III exhibits two distinctive absorption bands located at 350 and 395 nm, which can be attributed to the out-of-plane quadrupole and the out-of-plane dipole resonances, respectively^32^. The absorption peak at 350 nm corresponds to the SPR of the main body of the dendritic structure, and the peak at 395 nm possibly corresponds to the nano-sized secondary structure of FD III, with the feature size of 300–400 nm. With the increase of the treatment temperature, one can see that the intensity of all peaks decreases accordingly, and the peak at 395 nm decreases most significantly, which can be interpreted as the sintering and structural changes of the secondary structure, as shown in Supplementary Figs 14 and 15. However, FD VI shows three distinctive SPR absorption bands located at 350, 395, and 450 nm. As compared with those of FD III, the additional peak at 450 nm could be attributed to the in-plane quadrupole resonance, representing the tertiary structure sized ∼50 nm^33^. As the treatment temperature is raised to 80 °C, the peak at 450 nm disappears, corresponding to the disappearance of the tertiary structure due to the low-temperature sintering (Supplementary Fig. 16a–d). As the control sample, the commercial silver flakes show the main absorption peak at 350 nm, attributed to bulk silver^34^. As compared, the sintering process of silver flakes occurs at a higher temperature of ∼200 °C (Supplementary Fig. 17d). Moreover, as the temperature rises, the position of the main peaks for the silver flake control sample red-shifts, which can be attributed to the coarsened edges of silver flakes with the rise of temperature^35^. As compared, it can be found that the band of FD VI is the broadest, consisting with the wider size distribution range of FD VI. The above analysis suggests a distinctively low-sintering temperature of 80 °C of FD VI, which is benefited mainly by the numerous nanotip structures on the surface of FD VI.

The sintering processes of the powders of FD III, FD VI and 2D silver flake powders can be monitored by annealing at a series of temperatures ranging from 40 to 300 °C for 30 min in argon atmosphere. The thermal sintering mechanism is illustrated by Fig. 3d–g and the corresponding morphological changes of the FDs are shown in Supplementary Figs 15 and 16. The stepwise sintering process of FD VI can be summarized into three stages: the first stage takes place at 60 °C, when the tertiary structure begins to melt, and the silver grains coalesce with the secondary structure, making them thicker. The second stage occurs at 80 °C, when the tertiary structure disappears completely, and the secondary structure fuses into micro-silver plates, with an average diameter of 2–3 μm. In the third stage, as the temperature rises to 160 °C, those micro-silver plates transform to rod-like structures, indicating coarsening of the primary structure of FD VI; when the temperature further rises to 200 °C, the secondary structure disappears thoroughly, and the primary structure transforms into a round shape. As for FD III, the stepwise sintering process is similar to FD VI, but the corresponding temperature of each process is slightly higher (Supplementary Fig. 15). As a control, it can be found that the surface and the edge of silver flakes are smoothened at 250 °C (Supplementary Fig. 17), indicating the slightly sintering of the nanoscale surface features, which agrees well with the UV/vis absorption results. In summary, the sintering temperature of FD VI is much lower than that of FD III, and the morphology of FD VI can change significantly during the sintering process, indicating the better sintering ability of FD VI in low-temperature conditions. Furthermore, the FD-based conductive networks could maintain stable at 150–250 °C, as indicated in Supplementary Fig. 3.

### Study of the percolation characteristics of FD-ECCs

**Figure 4:**
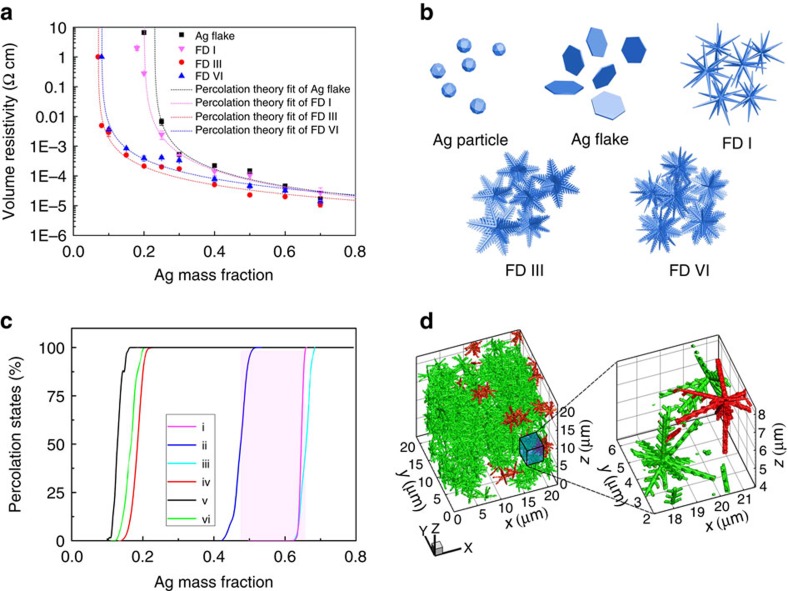
The volume resistivity of the FD-ECCs and the relevant percolating simulations. (**a**) A figure of the volume resistivity of the ECCs versus different silver filler contents (by mass) of various samples (FD I, FD III, FD VI and silver flake (control)); the dash lines represent the best-fit line for volume resistivity of various materials above percolation threshold using percolation theory. All ECC samples were cured at the temperature of 150 °C for 20 min. (**b**) the dispersion scenario of silver fillers within a transparent polymer matrix; (**c**) the simulated percentages of percolation status versus the applied Ag mass fraction for the five types of silver filler models: (i) spherical particle, (ii) flakes with random orientations, (iii) flake with shear alignment, and the pink zone represents for the percolation zone considering the alignment effect of the flake model; (iv) FD I, (v) FD III and (vi) FD VI; (**d**) a snapshot of the computed case consists of modelled FD III in Monte Carlo simulation, the left is a disposition of the FD III in simulation, and the right displays the magnified zone near a chosen spot with connected and isolated FD III particles. The percolating group is in green, the isolated groups are in red, which are not contributing to the percolation.

The above analyses show that FD III and FD VI have nearly symmetric 3D radial structure and low-temperature sintering ability (lower than 80 °C), both of which are beneficial to form electrical percolation network in the ECCs. In addition, the as-prepared FDs dispersed in ethanol can be robust enough to maintain integrity after a period of 30 min of sonication (ultrasound bath) with the power of 100 W (Supplementary Fig. 19). It assures that the FDs could maintain the 3D hierarchical structure after being mixed with epoxy resin during the preparation of the ECCs in a high-speed planetary rotary mixer. The improvement of electrical conductivity can be confirmed by testing the printed films based on the ECCs samples. The ECCs filled with FD III exhibit the highest electrical conductivity among those of the three types of fillers, as shown in Fig. 4a. In this curing condition, the edges of adjacent FD branches sinter with each other with the neck diameter of the fusing branches up to ∼100 nm, as shown in Fig. 3e–g, which is consistent with previous reports.

The electrical resistivities of the printed films of the ECC samples can be measured by the four-point probe method. As can be seen in Fig. 4a, the volume resistivities of FD I, FD III, FD VI and silver flakes-based ECCs are 2.73 × 10^−5^, 1.08 × 10^−5^, 1.40 × 10^−5^ and 1.64 × 10^−5^ Ω cm with the filler loading of 70 wt%, respectively. The volume resistivities of FD III and FD VI-based ECCs can be maintained at 5.08 × 10^−5^ and 7.80 × 10^−5^ Ω cm respectively, with the silver content of 40 wt%, which are similar to the silver flake-based ECCs with the content of 60 wt%. To be noted, the experimentally observed volume resistivities of the FD III-based ECC (FD III-ECC) with 8 wt% and the FD VI-ECC with 10 wt% are 4.9 × 10^−3^ and 3.6 × 10^−3^ Ω cm, respectively, which are the lowest filler contents above their percolation threshold. The volume resistivity of FD I-ECC with 20 wt% silver content is 9.7 × 10^−3^ Ω cm, which is significantly higher than both FD III and FD VI. Although the volume resistivity of silver flakes-based ECCs with 25 wt% is only 6.6 × 10^−3^ Ω cm; when the silver content is further decreased, higher and unstable resistivity can be obtained^25^. The cross-sectional SEM images of ECCs are shown in Supplementary Figs 20–22. The exponential decrease of volume resistivity of the FDs-based ECCs above the percolation threshold (dash lines in Fig. 4a, Supplementary Fig. 23 and Supplementary Table 6) can be observed, which is consistent with percolation theory^36^,^37^. To make a more detailed comparison, the resistivity data of the recently reported ECCs are summarized, including those based on Ag NPs, Ag NWs, Ag flakes, Cu NPs, Cu flakes and carbon materials (Supplementary Table 5). This comparison shows that the FD-ECCs possess a much better percolation probability than the Ag flakes-based counterparts, and their performances can be even better than most of the recently reported results based on other types of conductive fillers. For example, Wu *et al*.^38^ reported that the Ag nanoparticles mixed with UV-cured resin possessed the resistivity of 3.65 × 10^−1^ Ω cm with 56 wt% silver content. The single-walled carbon nanotube (SWCNT) based composite reported by Sekitani *et al*.^39^ showed a resistivity of 4.8 × 10^−4^ Ω cm with 15.8 wt% carbon nanotube (CNT) content. (To be noted, the density of SWCNT is about eight times lower than silver.)

### Study of the percolation mechanism of FD-ECCs

3D Monte Carlo simulation using the classic Hoshen–Kopelmen algorithm can give us a deeper insight into the percolation mechanism of these FDs^40^. FD I, FD III, FD VI, spherical silver particles and silver flakes were modelled and scattered into the simulation domain randomly for the observation of the percolation phenomena. Figure 4b indicates the various dispersions of the five different morphologies of the silver fillers (Supplementary Fig. 24 and Supplementary Table 7). As shown in Fig. 4d, the modelled FD III fillers are randomly positioned in a 3D simulation domain. The percolation probability represents the chance of developing a percolated network in the *x*-direction using a single-particle type, and the curves are drawn versus silver mass fractions shown in Fig. 4c. The resulting curves show that all FDs, regardless of the secondary features, critically percolate at ∼12.8–18.5 wt%, while those of the spherical particle (64.6 wt%) and silver flake (ranging from 47.5 to 66.0 wt%) models require much more silver content to percolate through the domain. To be noted, FD III reaches the lowest theoretical percolation threshold at ∼1.6 vol% (12.8 wt%), which is about 65% higher than the experiment observation (0.97 vol%). This discrepancy is a very small one, as even for the spherical fillers, a 65% volume difference corresponds to ∼18% diameter difference. Considering the much complicated 3D structure of FDs, the geometric difference is almost negligible. Nevertheless, to further improve the accuracy of simulation, some other factors such as the surface status of the FD fillers (such as surface energy and chemical treatment^25^) which can influence filler dispersity shall be considered.

### FD-ECCs for high-quality laser-scribe patterning

The unique low percolation threshold in a 3D space and low-temperature sintering properties render the FD-ECCs especially suitable for interconnects/circuits for flexible electronic products. From here after, we evaluate the process compatibility of the FD III-ECCs with laser-scribe patterning. Currently, laser-scribing technique is becoming the mainstream technique for fine circuit patterning for mobile electronics. For mobile electronics, the development of novel technique for the fabrication of flexible and ultra-thin-frame (for example, borderless smart phones) capacitive touch panel modules is urgently needed, as the shipment of global capacitive touch panel modules was over 1.5 billion pieces in 2014 and is still rising^41^. Even though conventional screen printing has been widely used for direct patterning of ECCs (silver pastes) to form fine circuits, it can hardly realize very fine circuit lines (for example, narrower than 50 μm) in a large scale. Moreover, to avoid shifting and coarsening of the circuit lines, repeatedly cleaning the screen mask is necessary after several printing cycles, which significantly reduces productivity. The leading solution in industrial sector is coupling the screen printing technique with the laser-scribing method (laser scribing fine patterns on a screen-printed raw profile), where the laser-scribing has pushed the line pitch down to 20 μm without compromising the high throughput and yield of the screen printing (see Supplementary Information). However, current laser-scribing technique still faces a few technical difficulties. For instance, because of the thermal stability mismatch of silver and resin, a higher laser power and multiple scribing are required to totally ablate the silver filler particles besides the resin, so as to guarantee yield, which, in turn, compromises the pattern resolution and the adhesion between printed circuit and substrate.

**Figure 5:**
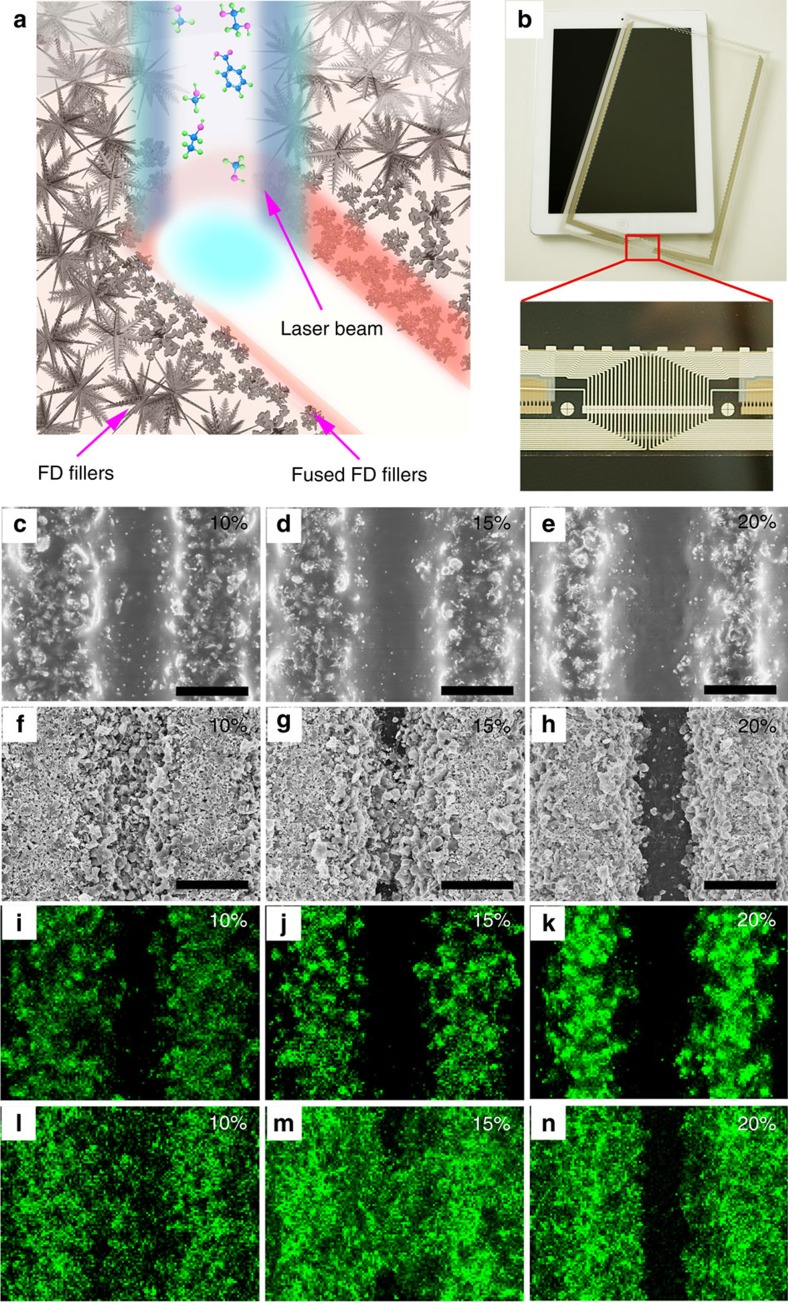
FD-ECCs for the laser-scribe circuit line patterning. (**a**) Schematic representation showing the laser beam scribing process for the FD-ECCs; the resin binder (polyester) is decomposed into small molecules and evaporated; (**b**) a photographic image of a capacitive touch panel module with the laser-patterned peripheral circuit lines for a pad; (inset: the detailed morphology of the laser-scribed circuits.) (**c**–**e**) SEM images of laser-scribed grooves of FD III-ECC with the laser-scribing power of 10, 15 and 20%; (**f**–**h**) SEM images of the laser-scribed grooves of commercial silver paste (FTL-630LE) with the laser-scribing power of 10, 15 and 20%; (both ECCs samples are screen printed and cured on PET; scale bars, 20 μm). (**i**–**k**) EDS mapping images of silver elemental distribution, corresponding to (**c**–**e**); (**l**–**n**) EDS mapping images of silver elemental distribution, corresponding to (**f**–**h**).

Here we demonstrate that the unique low temperature fusing cut-off and low filler loading characteristics of the FD-ECCs promise them an excellent motif for laser-scribing fine circuit patterning, as compared with the conventional silver pastes. As illustrated in Fig. 5a, when laser beam passes through, the resin binder in the FD III-ECC is immediately decomposed and evaporated; meanwhile, the conductive network can be disconnected due to rapid fusing and shrinkage of the FD III fillers, rendering highly efficient etching effect with much lower laser power. To avoid unnecessary etching of the film substrate (that is, polyethylene terephthalate, PET, see Supplementary Fig. 25) and to be consistent with conventional practice, a wavelength of 1,064 nm was selected, and the ablated line width/distance was controlled to be 20 μm. Taking a close look at the laser-scribed FD III-ECC patterns (Fig. 5c-e), those circuit lines exhibit a much cleaner opened path than the commercial benchmark ECC-based ones (Fig. 5f–h). Even though the edges of the patterned FD III-ECC lines exhibit a zig-zag morphology, the distribution of Ag element remained in straight lines within the resin matrix, suggesting excellent linearity of the laser-scribed patterns (Fig. 5i-k for SEM-EDS mapping result). Moreover, those ablated FD III filler particles distributed at the rim of the patterned lines exhibited the similar round shape to the thermally sintered samples as shown in Fig 3g. For comparison, FD III-ECC can be laser-scribed with much lower laser power (only about 1/2 of the power) than that of the commercial control sample. FD III-ECC possesses similar electrical resistivity (2–3 × 10^−4^ Ω cm) to the commercial control ECC after the laser-scribing process, which can well cater to the current market need (see Supplementary Fig. 27). More detailed optical images and 3D morphology images are shown in Supplementary Figs 28 and 29. The mechanical properties of the FD-based ECCs can be verified by the tape test and the bending test, as shown in Supplementary Figs 30 and 31, which indicate that the FD-based ECCs has better adhesive to the substrate. Moreover, the thermal-humidity reliability test results can prove that the FD-based ECCs had sufficient reliability for practical applications (as shown in Supplementary Fig. 32). The mechanism of laser etching on the FD-based ECCs is discussed in Supplementary Information, shown as Supplementary Fig. 33. Thereafter, the new technology can provide better ECC to substrate binding strength, lower materials cost and energy budget, and is more compatible to thinner plastic substrates, which are all features as the essential requirement for the future rollable displayers, super high-frequency mini-antennas and other novel device techniques.

## Discussion

In summary, we for the first time demonstrate the scalable synthesis of narrow size distribution FDs with the tailored 3D micro-/nanostructures, and systematically reveal the unique low-temperature sintering mechanism and percolation mechanism of the FDs as conductive fillers in composites. These FDs featured with abundant nano-sized tips are the key to the excellent low-temperature sintering property (<80 °C) so as to form a conductive network efficiently. Experimental results demonstrate that the percolation threshold for FD III-ECC is 0.97 vol% (8 wt%) and for FD VI-ECC is 1.03 vol% (8.5 wt%), which are confirmed by Monte Carlo simulation results. Because of their unique low-temperature sintering property and fusing cut-off characteristic, the FD-ECCs have extended the laser-scribe patterning capability to 20 μm line space with ∼50% reduction in laser power requirement. The present work shows a great promise in the fine flexible circuit patterning applications.

## Methods

### Synthesis of FDs

The supplying rate of Ag^+^ and the reducing agent were accurately controlled, so as to critically control the morphology, sizes and reproducibility in a scalable preparation scheme (Supplementary Fig. 1a). Typically, 2 l of AgNO_3_ (0.06 M) and NH_2_OH (Alfa Aesar) (0.24 M) aqueous solutions were continuously pumped at the same velocity and mixed together by peristaltic pump. The droplets are mixed in the conical flasks array, which were shaken gently at room temperature on the orbital shaker. The precipitate was collected and washed by deionized water for three times, and then dried in a vacuum desiccator at room temperature.

### Preparation of ECCs

Four types of silver powders were selected as conductive fillers, including FD I, FD III and FD VI, and a control micro-silver flake (Chengdu Banknote Printing Complex, product No. SF-01A, sized 5.0 μm, D90: 3.8–5.8 μm). They were treated by iodine solution to improve the electrical conductivities and the optimized iodine concentration were 4 wt% for micro-silver flake, 4 wt% for FD I, 6 wt% for FD III and 8 wt% for FD VI, respectively^20^. Afterwards, the treated silver powders were mixed with bisphenol-A type epoxy (Shell Epon 828) epoxy resins and methyl tetrahydrophthalic anhydride (MTHPA, Lindau Chemicals) (1:1 by mole ratio based on the epoxide equivalent weight of the epoxy resin and the hydroxyl equivalent weight of the hardener) in a planetary rotary mixer (Hasai Co, Shenzhen, China) at 1,500 r.p.m. for 10 min. Hexamethylenetetramine was used as catalyst with the concentration of 0.5 wt% to the resin. The silver contents of different ECCs were in the range from 5 to 70 wt%.

In addition, the ECCs samples (both FD III-ECC (polyester-based resin, 50 wt% silver content) and commercial silver paste (polyester-based resin, 75 wt% silver content)) were screen printed on PET and laser scribed (Fig. 5b). The conductivities of the patterned samples were measured on a Wafer Probe Station. Commercial silver paste (FTL-630LE, FP Co Ltd) was used as a control.

### Characterizations

The morphologies of FDs (FD I, FD III and FD VI) were studied by field emission SEM (HITACH S4800, Japan) and TEM (FEI tecnai G2 F30). Meanwhile, the sintering processes of the FDs and the cross-sectional images of ECCs samples were observed by SEM as well. The crystal properties of the FD III, FD VI and silver flakes were recorded by powder X-ray diffraction using a Rigaku diffractometer (D/MAX-2500, Japan) equipped with Cu-Kα radiation (*λ*=1.5418 Å). The surface species and chemical states were characterized by the X-ray photoelectron spectroscopy (XPS, ESCALAB 250Xi) measurement. Raman spectra were recorded by a Jobin-Yvon Horiba 800 spectrometer using argon laser source (532 nm). Thermal gravimetric analysis/differential scanning calorimetry analysis was carried out on a thermogravimetric analyser (NETZSCH SAT 449F3, Germany). The light absorption properties of the FDs were characterized by UV/Vis spectroscopy (SCINCO, S-4100, Korea) with an integration sphere and the scanning wavelength was from 200 to 1,000 nm. The nitrogen adsorption/desorption data were recorded at the liquid nitrogen temperature (77 K) using a 30 Micromeritics ASAP 2020 C apparatus. The samples were degassed at 120 °C under vacuum for 3 h before the measurement. The specific surface area was calculated using the Brunauer–Emmett–Teller equation. Total pore volumes were calculated from the amount adsorbed at a relative pressure (*P/P*_*0*_) of 0.99.

For electrical resistivity measurement, the ECC pastes were doctor-bladed onto a piece of glass slide, using two pieces of scotch tapes as the confinement; the gap between the tapes was precisely controlled into 2 mm and the length of printed ECCs was 30 mm. The ECCs with different silver contents were cured for 20 min at 150 °C. Five specimen of each sample were measured. The resistivity *ρ* was based on the follow equation:


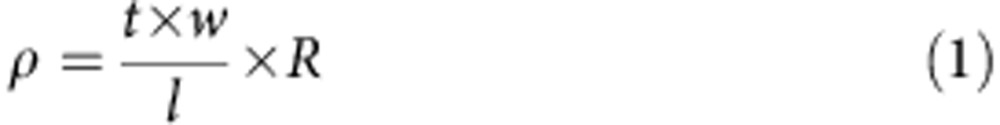


where *t*, *w* and *R* are the thickness, width and resistance, respectively.

The laser-scribe pattern processing was carried out on an Ag Laser Etching System (StrongLaser Co, laser wavelength: 1,064 nm, straight line processing speed: 1,000 mm s^−1^) with the maximum laser power of 20 W (Supplementary Fig. 26). The optical microscope images were obtained on a KH-7700 3D video microscope. The Reliability test (85 °C /85 RH) analysis of the ECCs samples were carried out in an ESPEC SETH-Z-042L humidity chamber.

## Additional information

**How to cite this article:** Yang, C. *et al*. Fractal dendrite-based electrically conductive composites for laser-scribed flexible circuits. *Nat. Commun*. 6:8150 doi: 10.1038/ncomms9150 (2015).

## Supplementary Material

Supplementary Information Supplementary Figures 1-33, Supplementary Tables 1-7, Supplementary Discussion and Supplementary References
