# Supplementary material for: Fractal dendrite-based electrically conductive composites for laser-scribed flexible circuits
Source: Nat Commun. 2015 Sep 3;6:8150. doi: 10.1038/ncomms9150 (PMC4569727; doi:10.1038/ncomms9150)
Supplement: Supplementary Information — Supplementary Figures 1-33, Supplementary Tables 1-7, Supplementary Discussion and Supplementary References [file ncomms9150-s1.pdf]

Supplementary Figures

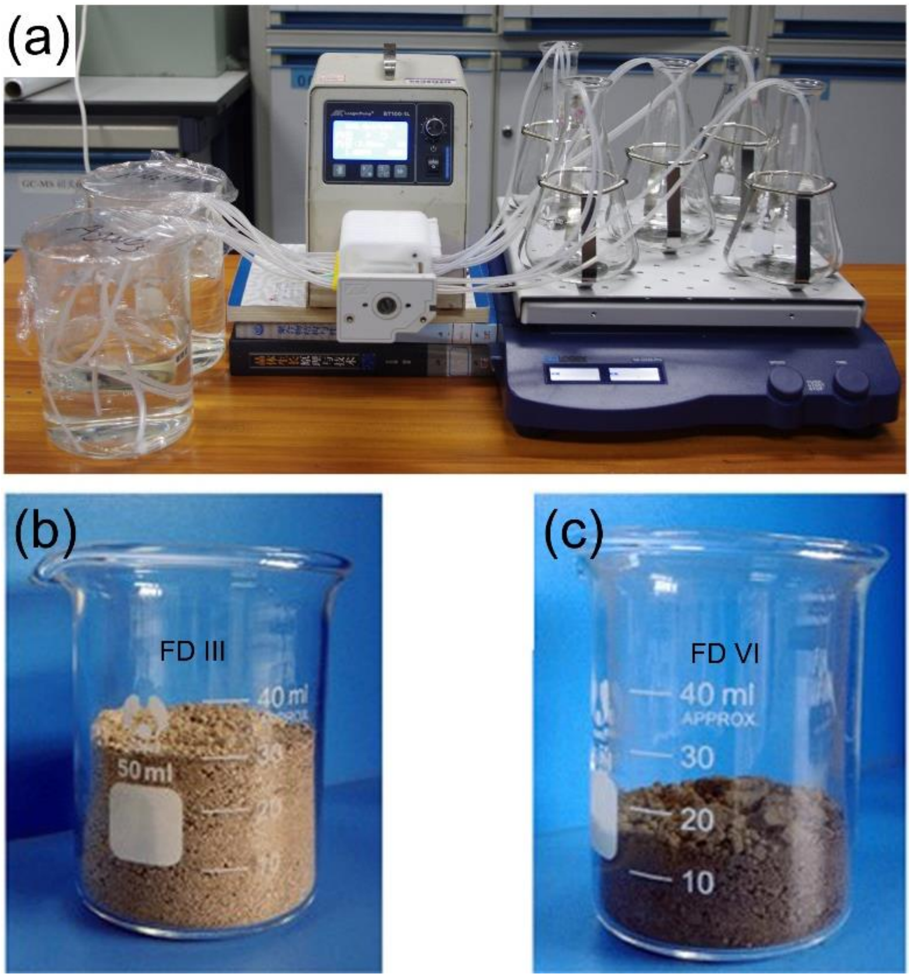

Supplementary Figure 1. (a) Photo picture of the micro-droplet reaction system for multiple channels scenarios; (b) and (c) are photographic images of the as-synthesized FD III and FD VI, respectively.

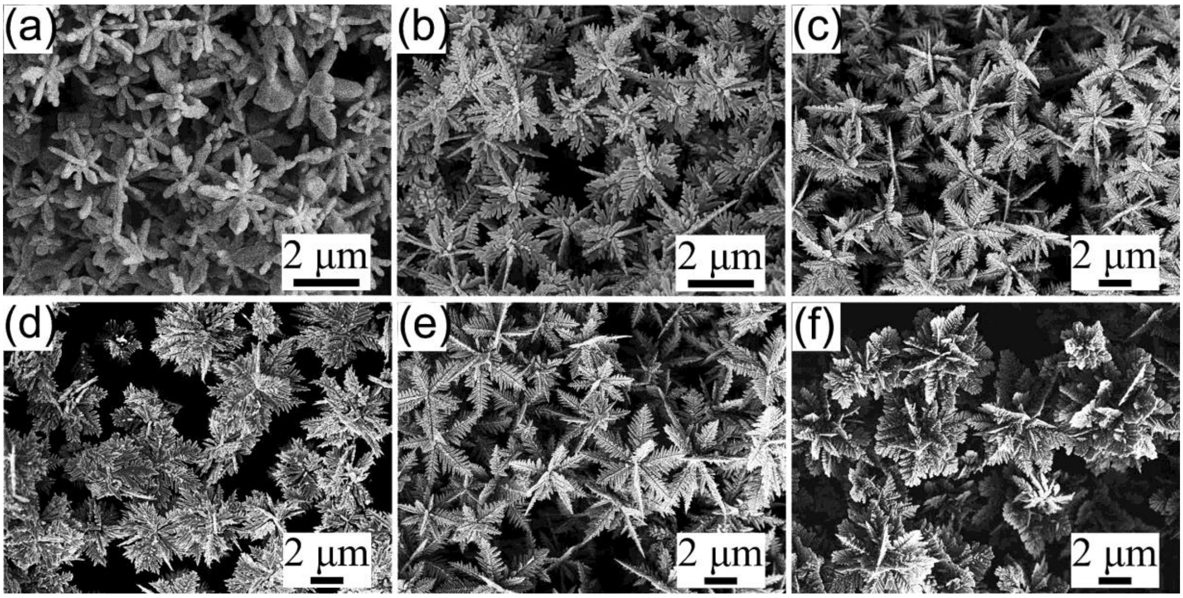

Supplementary Figure 2. SEM bird view images of the FDs corresponding to Fig. 1a. (a) FD I, size: 1.5-2.5 μm, (b) FD II, size: 1.8-3 μm, (c) FD III, size: 3-5.5 μm, (d) FD IV, size: 3.5-6.5 μm, (e) FD V, size: 5-7 μm, and (f) FD VI, size: 5.5-7.5 μm.

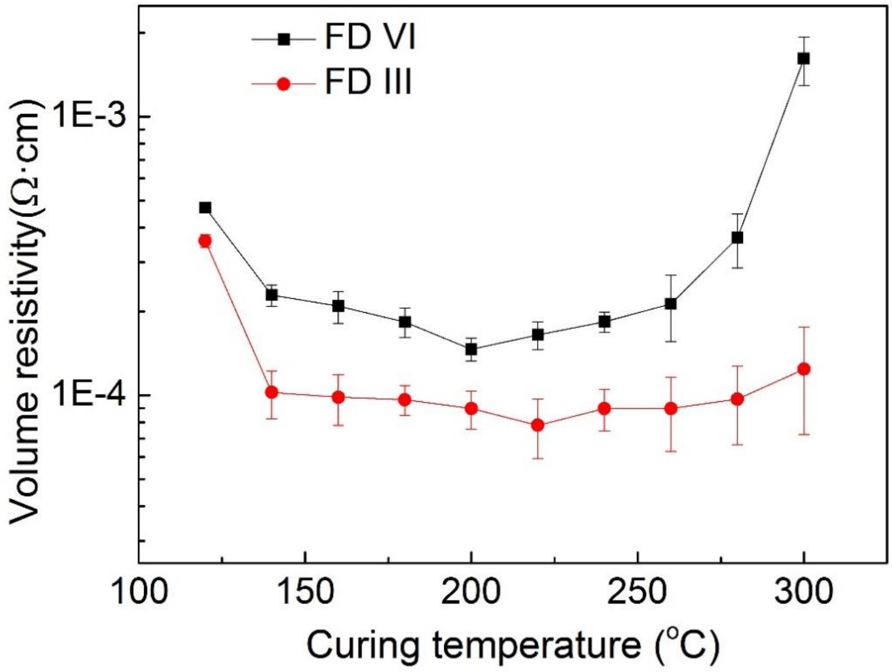

Supplementary Figure 3. The volume resistivity of FD-based ECCs changed with the increasing curing temperature.

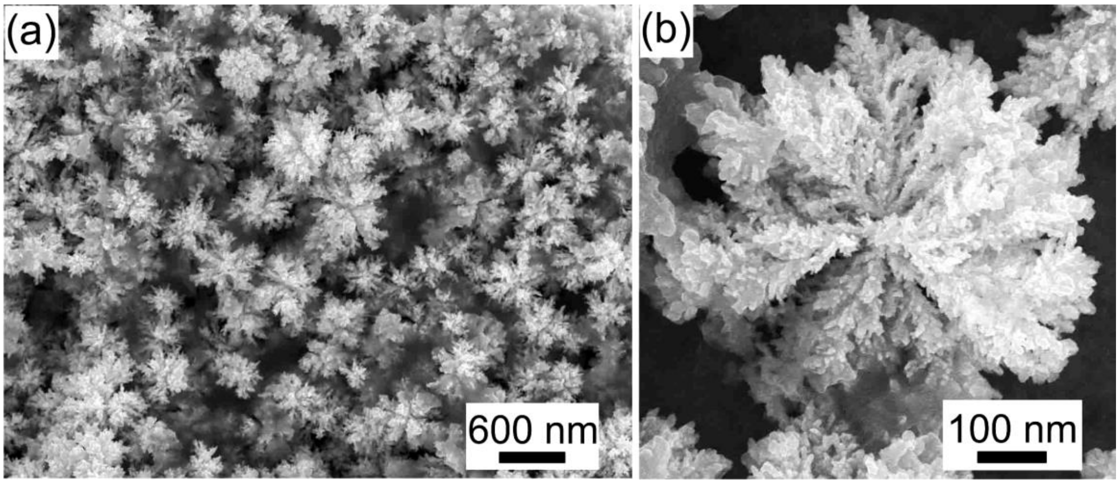

Supplementary Figure 4. (a) SEM image of the 3-D fractal Au micro-dendrites; (b) magnified image of the same sample.

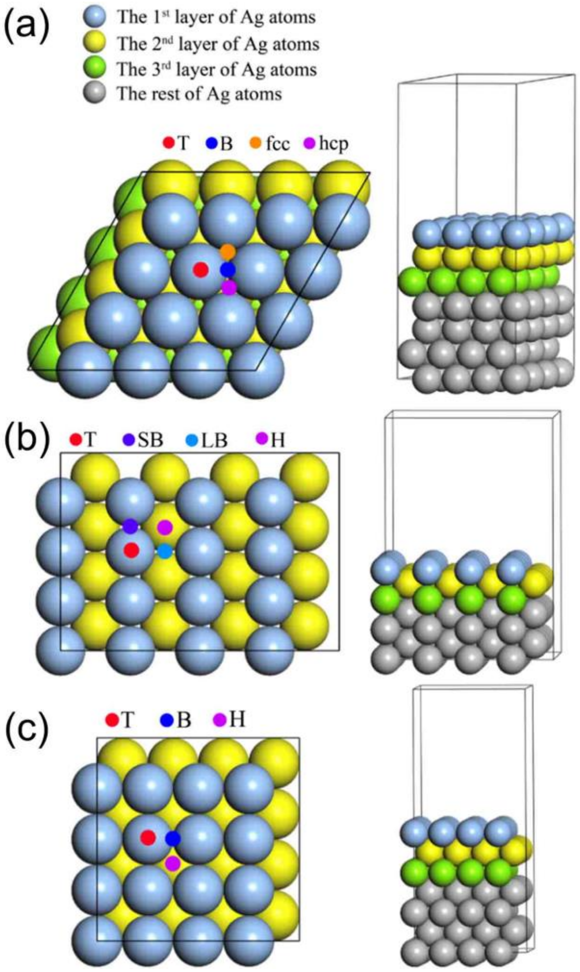

Supplementary Figure 5. Schematic top (left) and side (right) views of 4 × 4 (a) Ag (111), (b) Ag (110) and (c) Ag (100) surface.

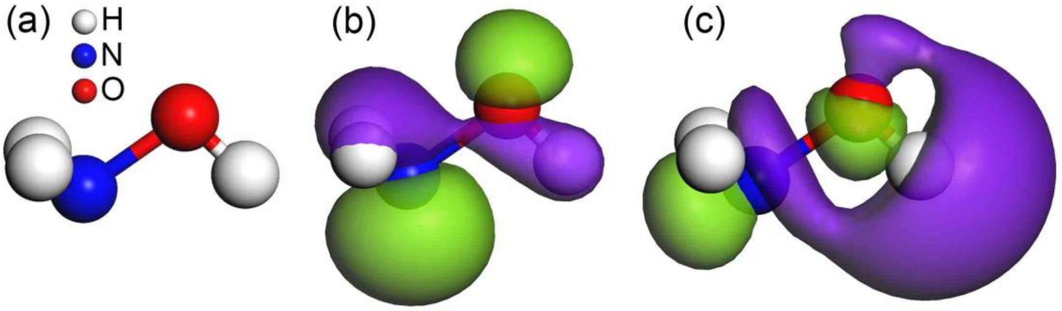

Supplementary Figure 6. Three dimensional views of (a) optimized structure, (b) HOMO orbital, and (c) LUMO orbital of the isolated NH_2OH molecule.

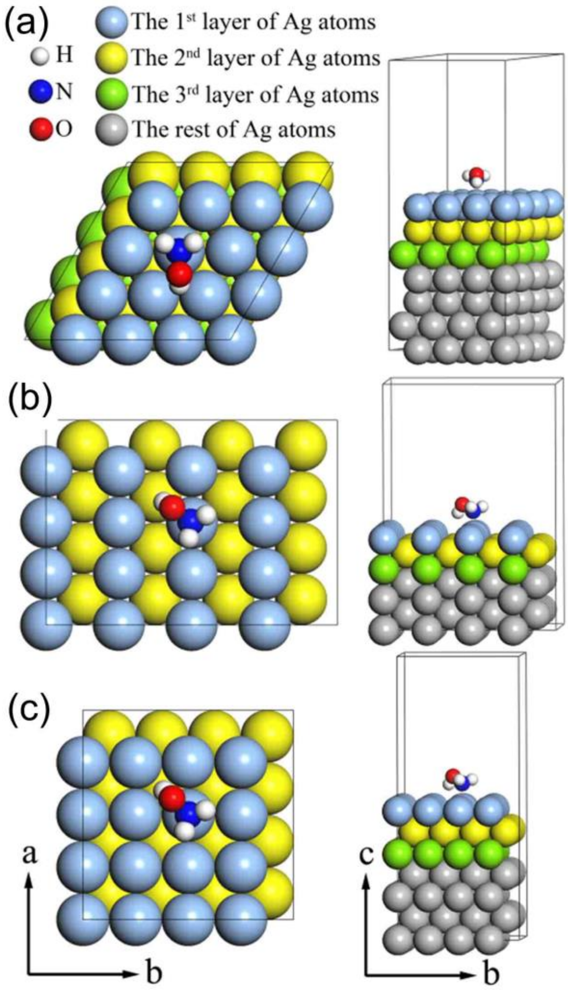

Supplementary Figure 7. Top (left) and side (right) views of optimal structures of NH_2OH adsorbed sites (4 × 4), (a) Ag (111), (b) Ag (110) and (c) Ag (100) surfaces.

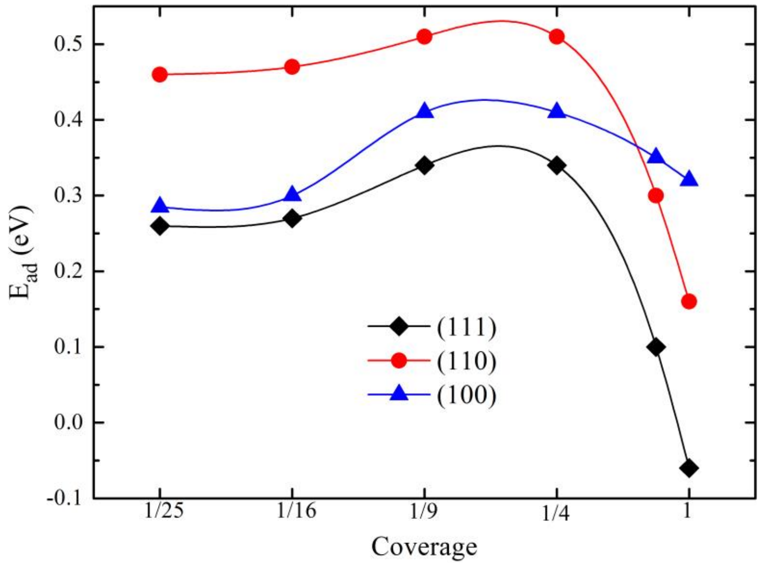

Supplementary Figure 8. Adsorption energy (E_{ad}) for NH_2OH adsorption on top sites of Ag surfaces with varying coverage of molecules.

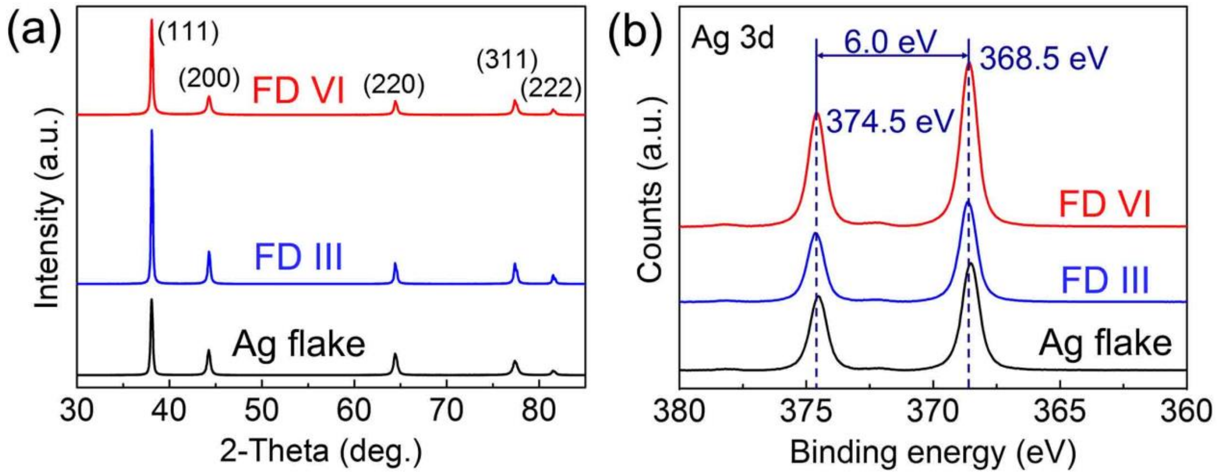

Supplementary Figure 9. (a) XRD pattern of FD VI (red), FD III (blue) and Ag flakes (dark); (b) XPS data of FD VI (red), FD III (blue) and Ag flakes (dark).

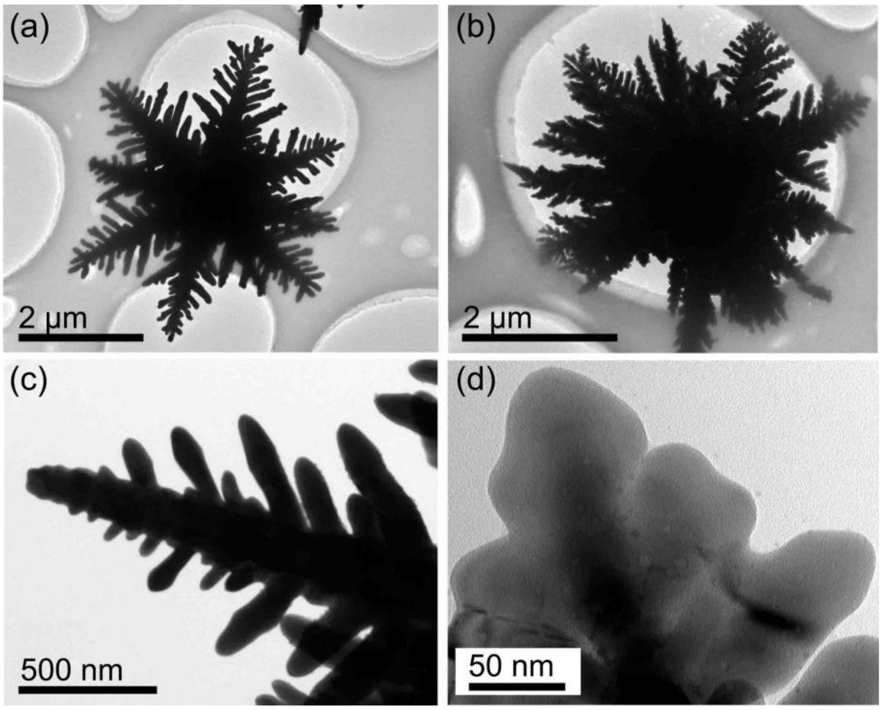

Supplementary Figure 10. TEM images of (a) FD III and (b) FD VI; (c) and (d) are the magnified images of (a) and (b), respectively.

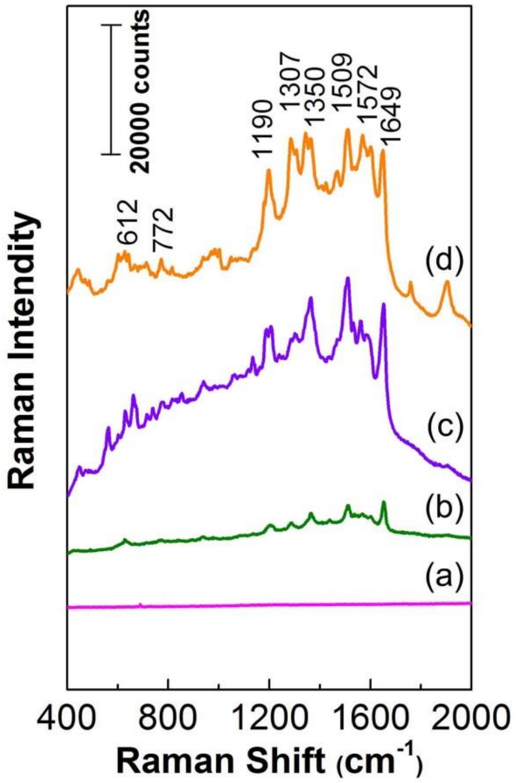

Supplementary Figure 11. Raman analysis of the Rhodamine 6G sample (1×10^{-6} M) on different substrates: (a) glass slide, (b) Ag flake, (c) FD III, (d) FD VI.

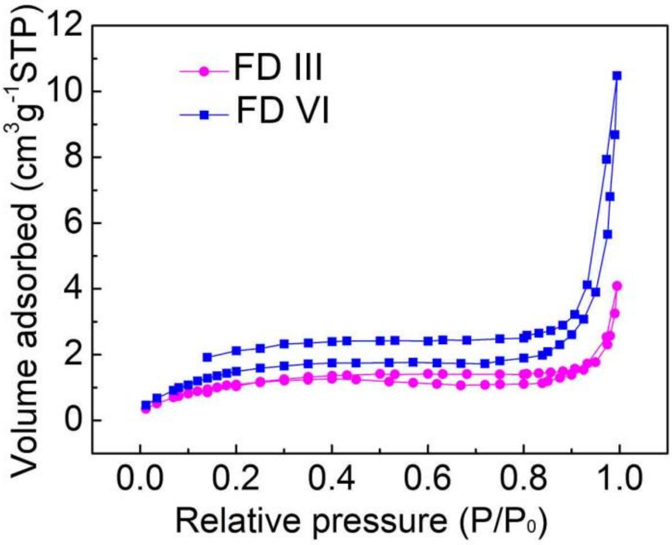

Supplementary Figure 12. Nitrogen sorption isotherms of FD III and FD VI.

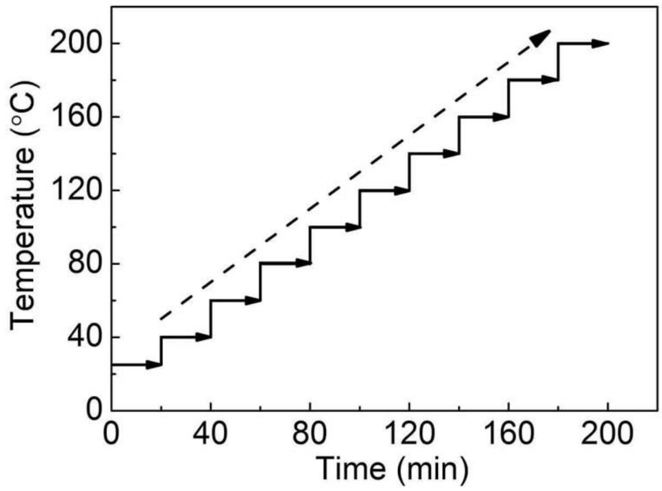

Supplementary Figure 13. Temperature-time curve of the heating process, which corresponds to the testing condition for the experimental results shown in Fig. 3.

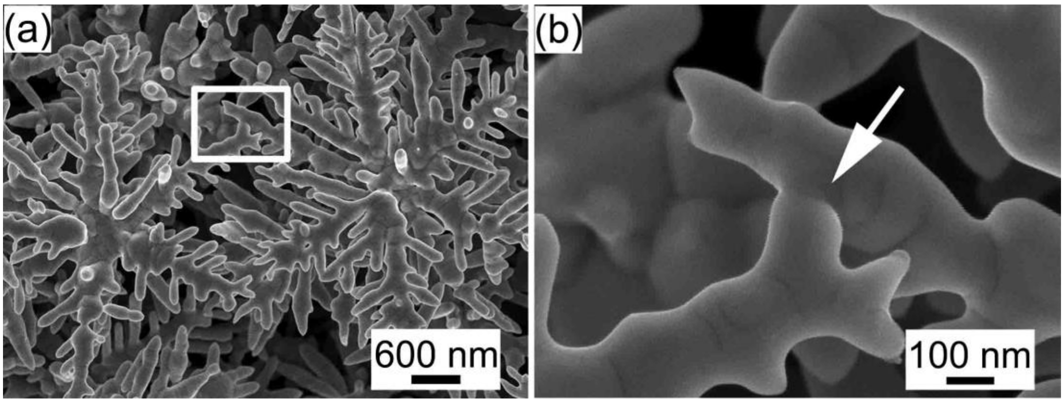

Supplementary Figure 14. (a) SEM image of the sintered FD III after heating at 150 °C for 30 min; (b) magnified image of the local microstructure in (a).

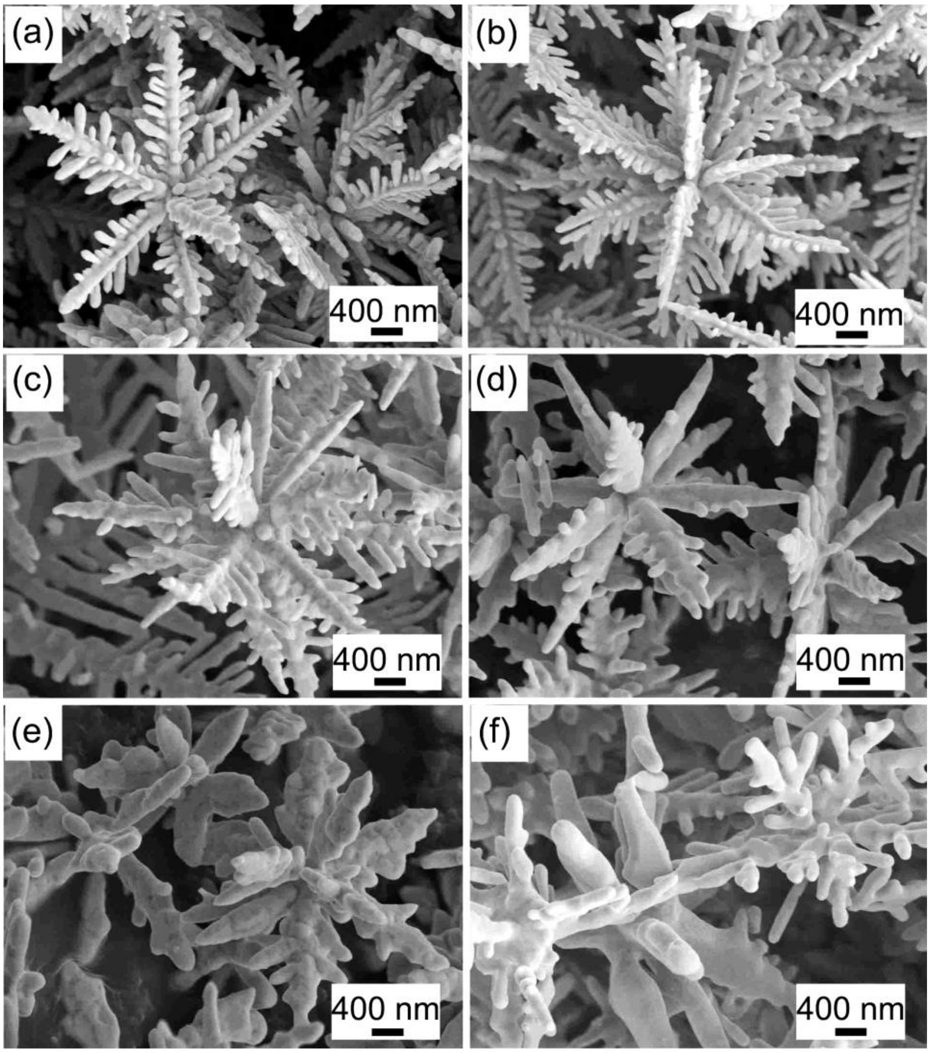

Supplementary Figure 15. SEM images of FD III after annealing at the temperature of (a) to (f) are 60 °C, 100 °C, 150 °C, 200 °C, 250 °C and 300 °C for 30 min in argon atmosphere.

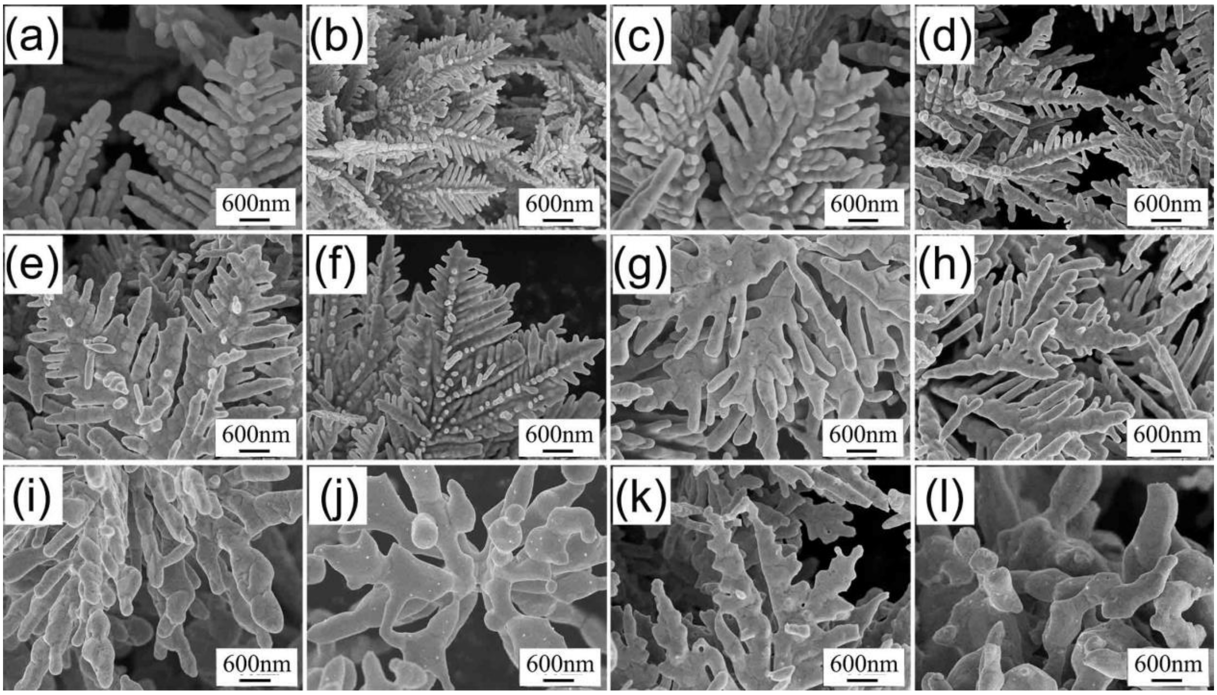

Supplementary Figure 16. SEM images of FD VI after annealing at the temperature of (a) 40 °C, (b) 60 °C, (c) 80 °C, (d) 100 °C, (e) 120 °C, (f) 140 °C, (g) 160 °C, (h) 180 °C, (i) 200 °C, (j) 220 °C, (k) 250 °C and (l) 300 °C for 30 min in argon atmosphere.

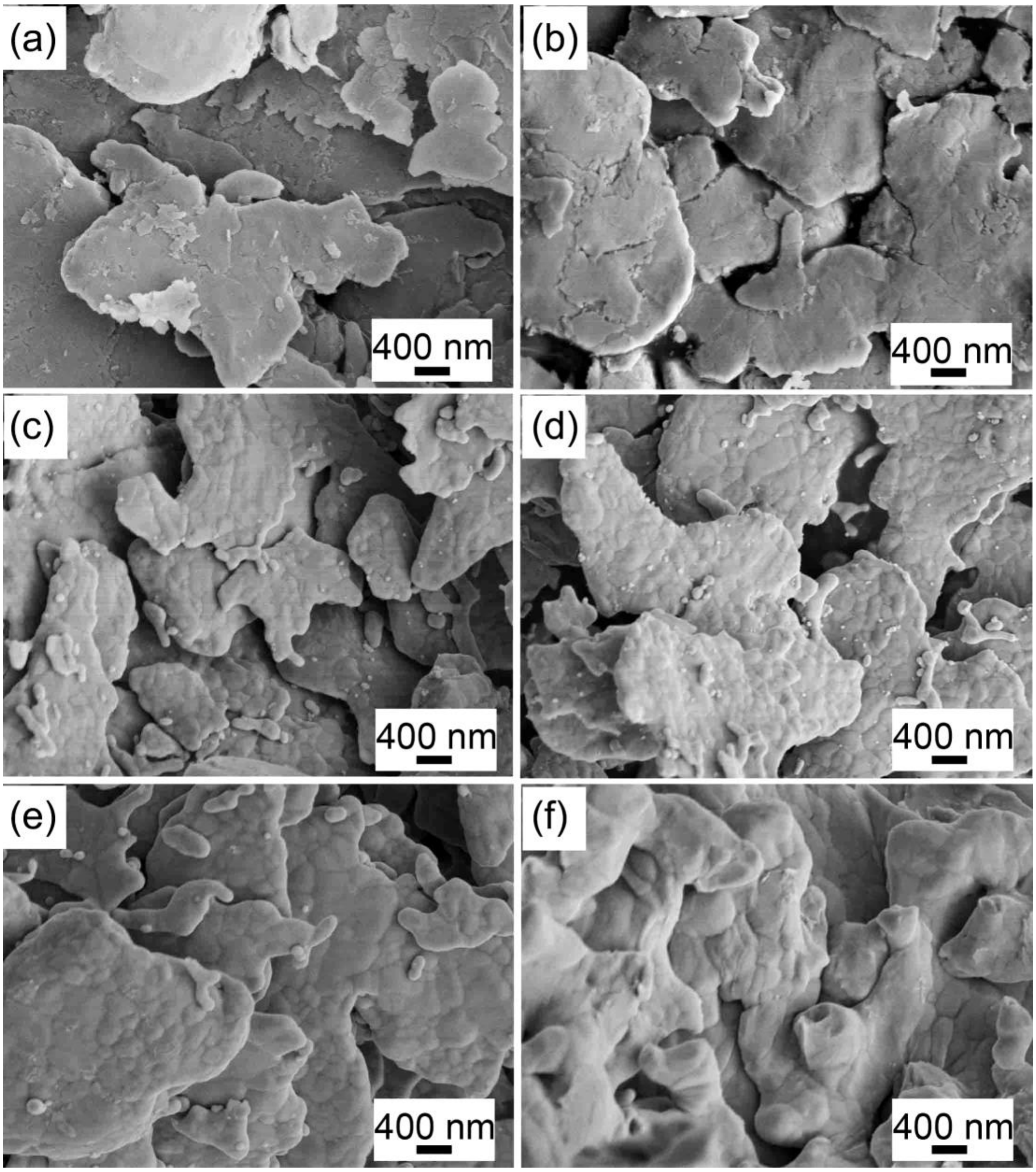

Supplementary Figure 17. SEM images of Ag flake after annealing at the temperature of (a) to (f) are 60 °C, 100 °C, 150 °C, 200 °C, 250 °C and 300 °C for 30 min in argon atmosphere.

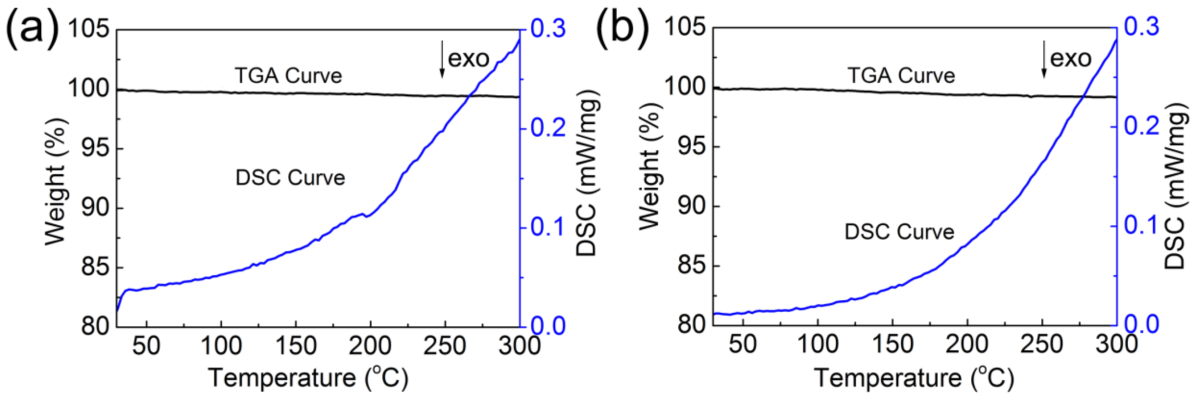

Supplementary Figure 18. TG/DSC analysis of (a) FD III and (b) FD VI. The samples were ramped from 25 °C to 300 °C in argon at 2 °C min^{-1}.

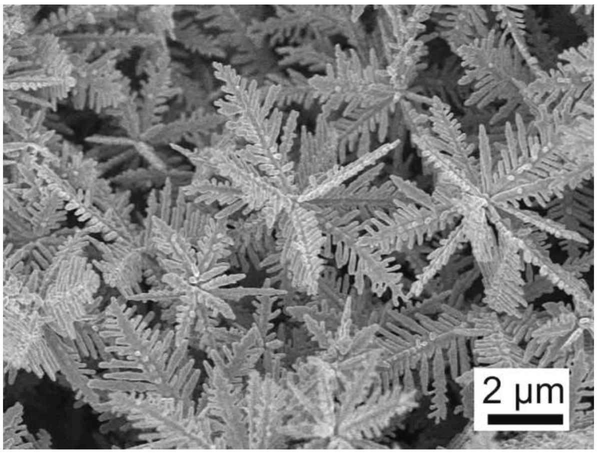

Supplementary Figure 19. SEM image of FD III after 30 min, 100 W sonication, the 3-D fractal structure kept intact.

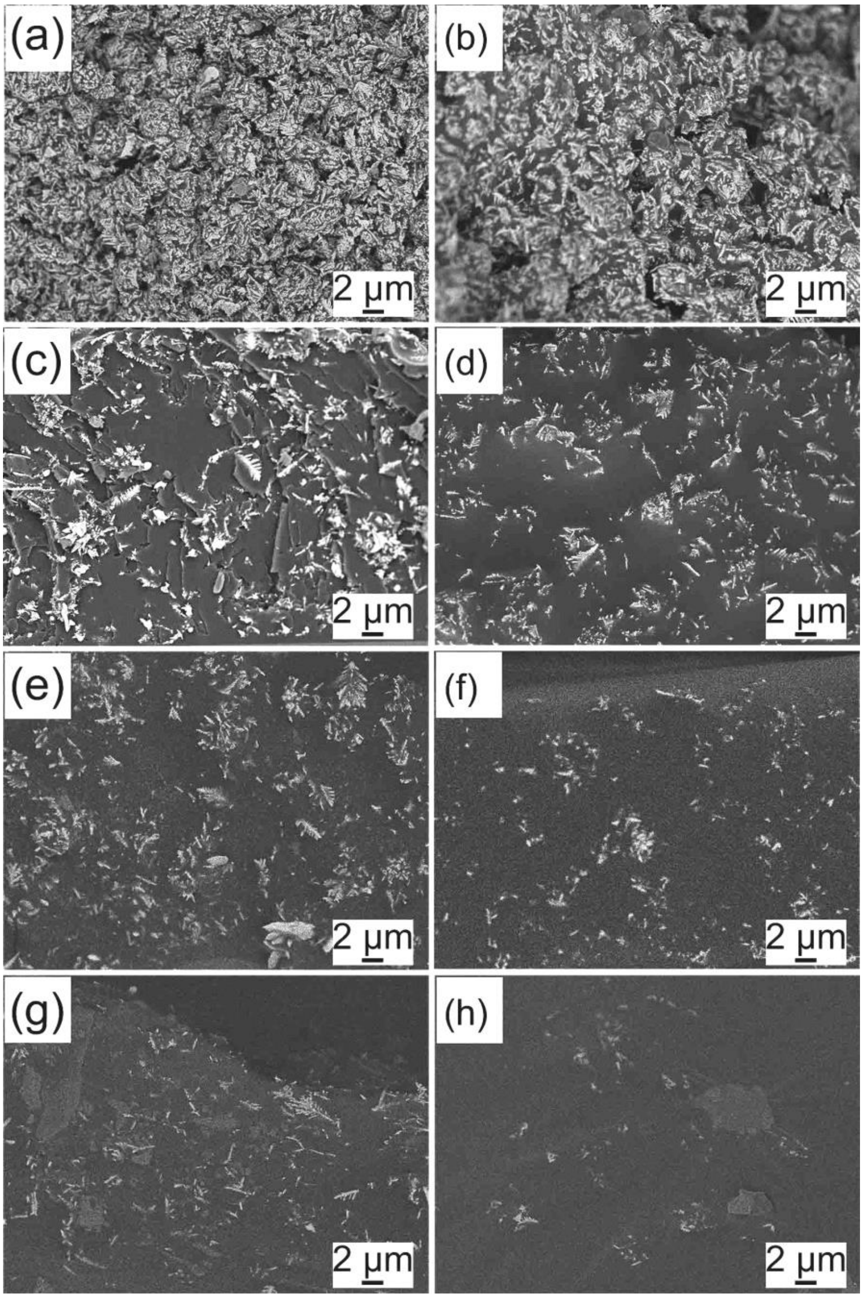

Supplementary Figure 20. Cross sectional images of ECCs with different silver content (FD VI) by SEM: (a) to (h) are 70 wt%, 60 wt%, 50 wt%, 40 wt%, 30 wt%, 20 wt%, 15 wt% and 10 wt%, respectively.

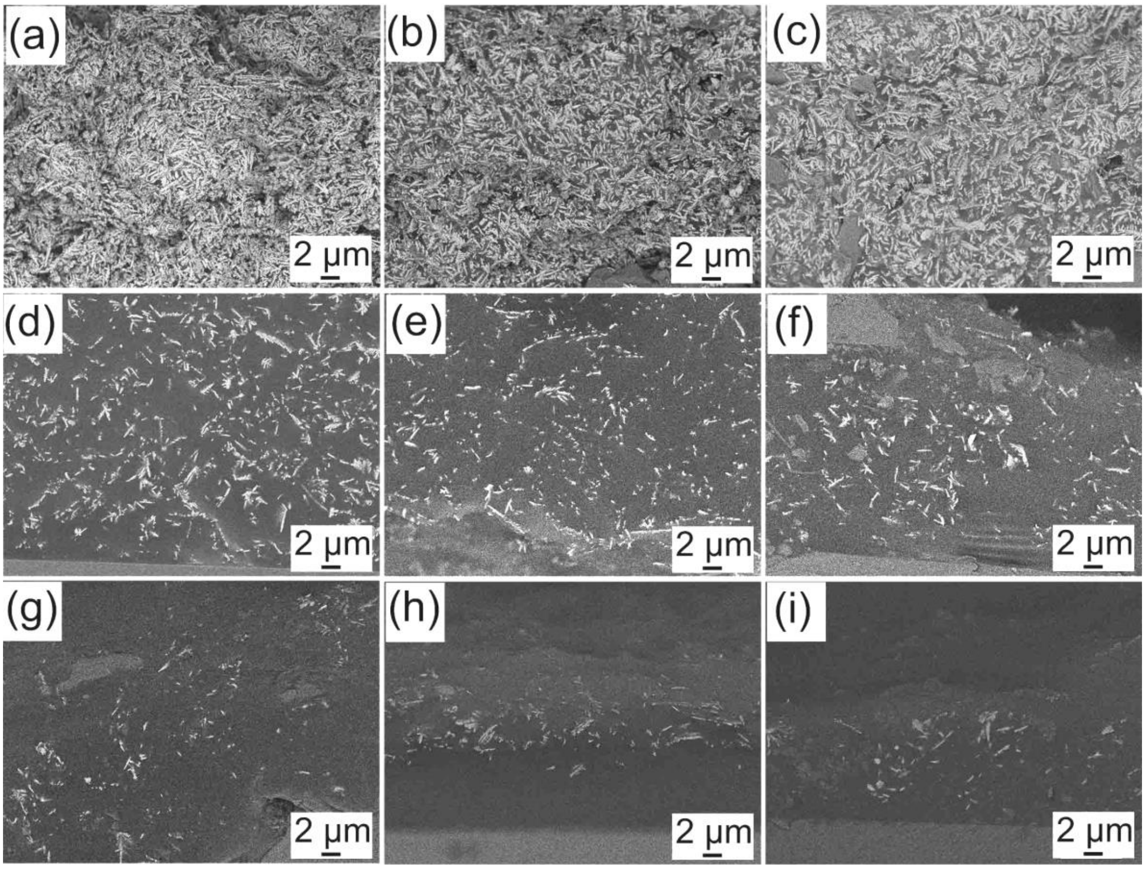

Supplementary Figure 21. Cross sectional images of ECCs with different silver filler (FD III) content by SEM: (a) to (i) are 70 wt%, 60 wt%, 50 wt%, 40 wt%, 30 wt%, 20 wt%, 15 wt% ,10 wt% and 8 wt%, respectively.

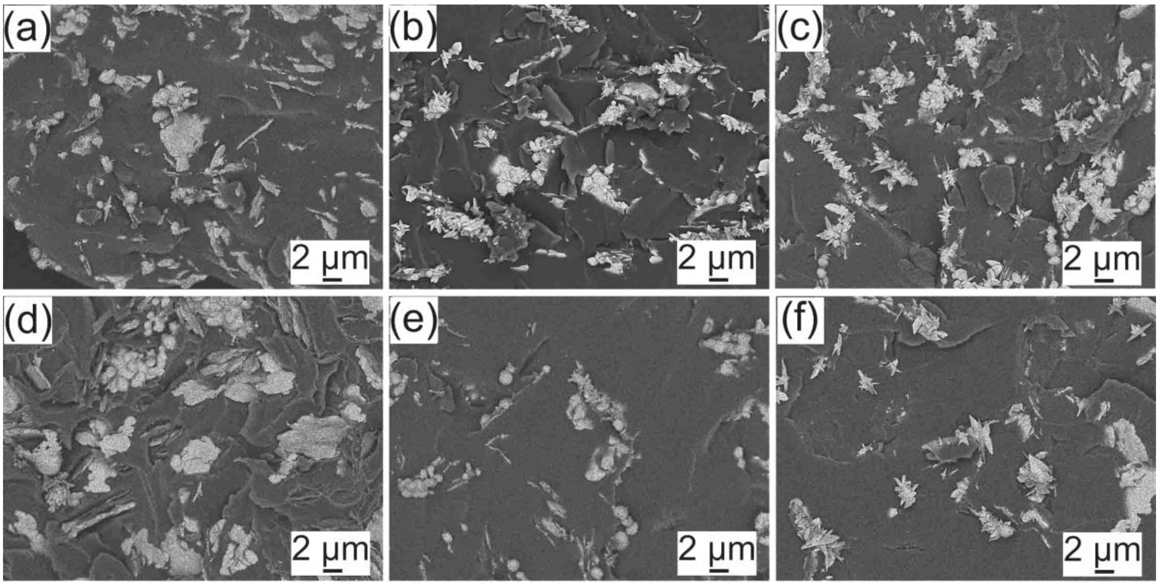

Supplementary Figure 22. Cross sectional images of ECCs with different silver content of Ag flake by SEM: (a) to (f) are 70 wt%, 60 wt%, 50 wt%, 40 wt%, 30 wt% and 25 wt%, respectively.

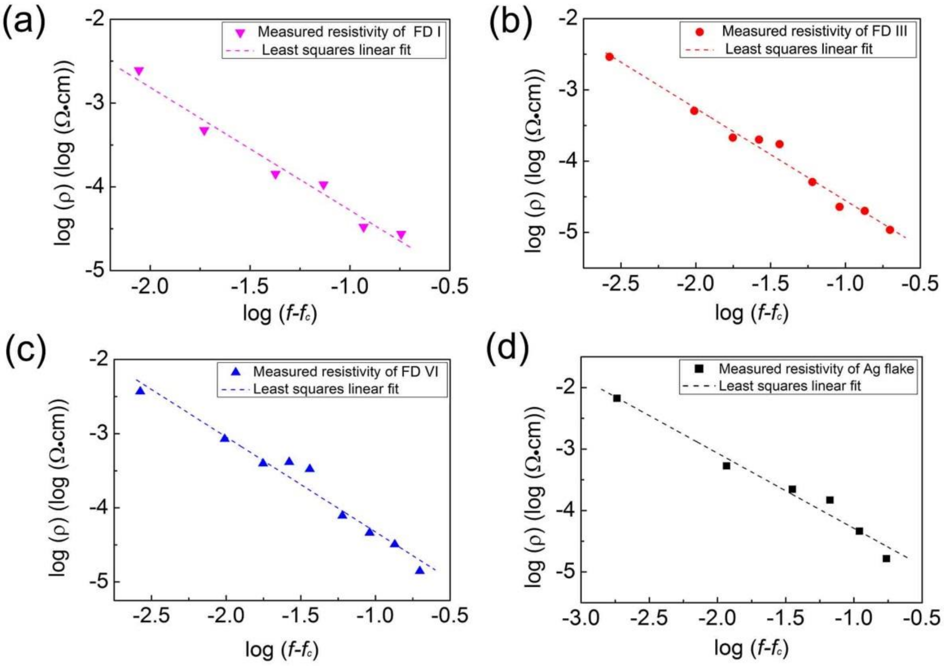

Supplementary Figure 23. Linear least squares regression fit of the measured resistivity versus mass ratio of Ag with (a) FD I, (b) FD III, (c) FD VI and (d) Ag flake (control sample).

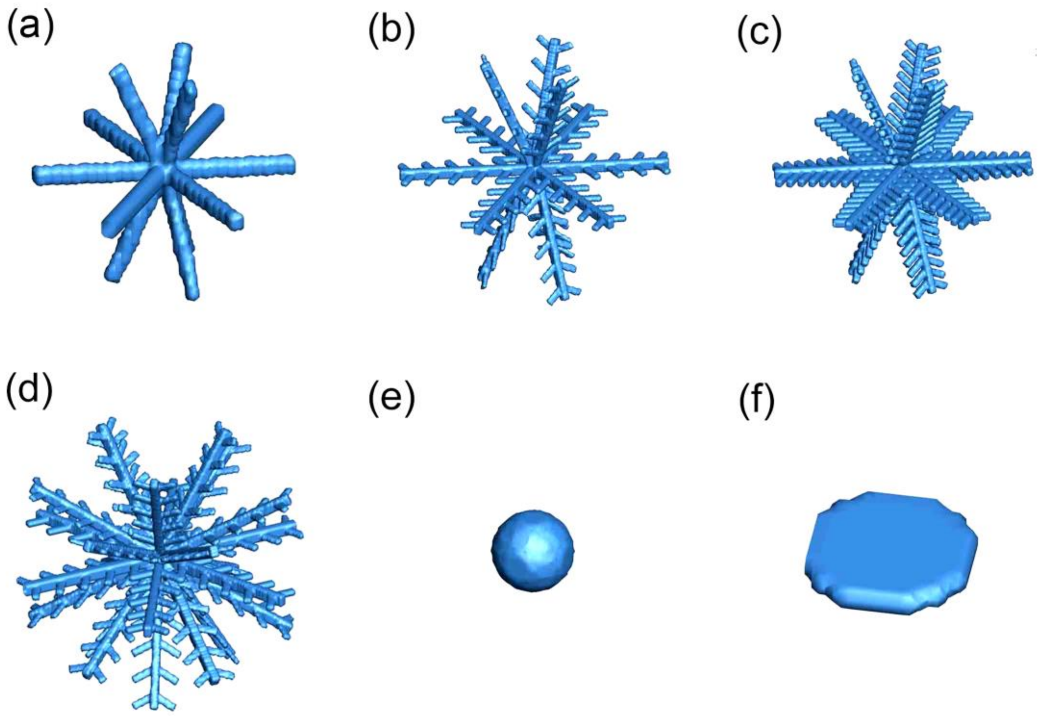

Supplementary Figure 24. (a)-(f) are some of the crystal models used for the 3-D Monte Carlo computation. (a) is a 12-branch particle for modeling FD I; (b) and (c) are the 12-branch FD III models with different secondary branch

density, (d) is an approximation model of the 24-branch FD VI, which has a better angular spatial distribution than FD III model, but consumes more silver. Any tertiary fractual features are ignored so as to improve the computation efficiency; (e) is a featureless sphere to serve as an approximation model of regular polyhedral silver crystal. (f) is used as a model for Ag flake. In the L^3 volume, the silver occupied space constribute to the silver volume fraction, their respective geometrical dimensions are listed in Supplementary Table 7.

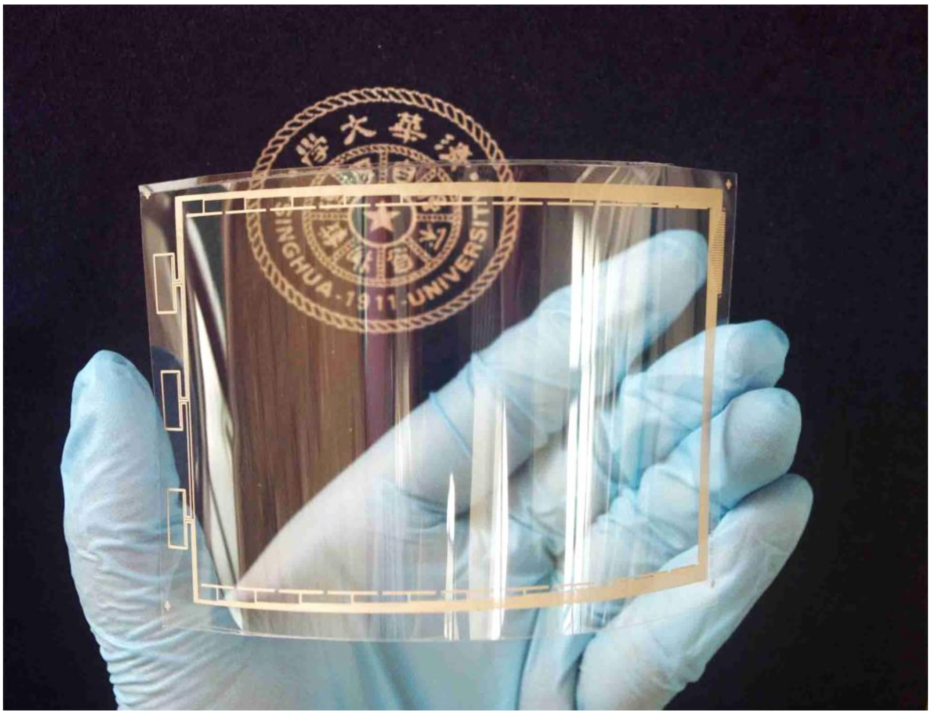

Supplementary Figure 25. Photographic image of a piece of ECC-screen printed film on PET for touch panel module.

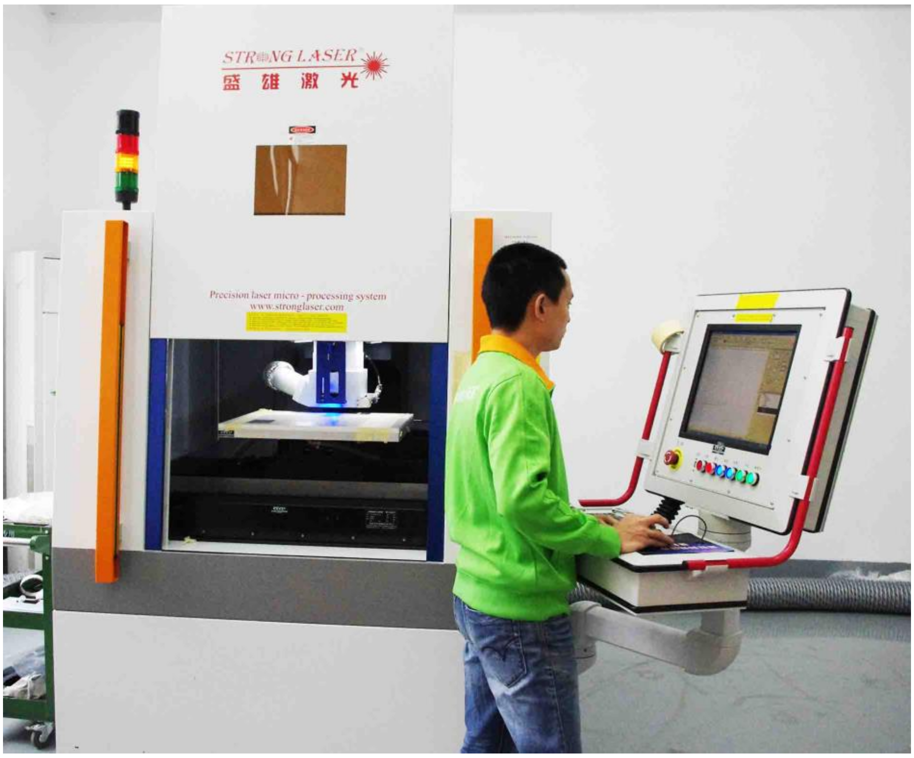

Supplementary Figure 26. Photographic image of the laser micro-processing system for use.

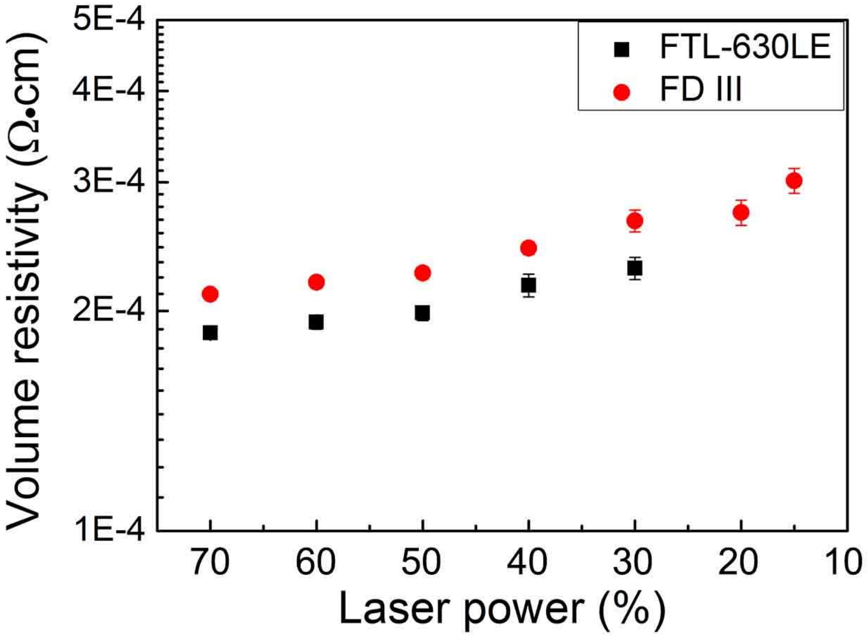

Supplementary Figure 27. Volume resistivity of the ECCs as a function of laser-scribing power levels.

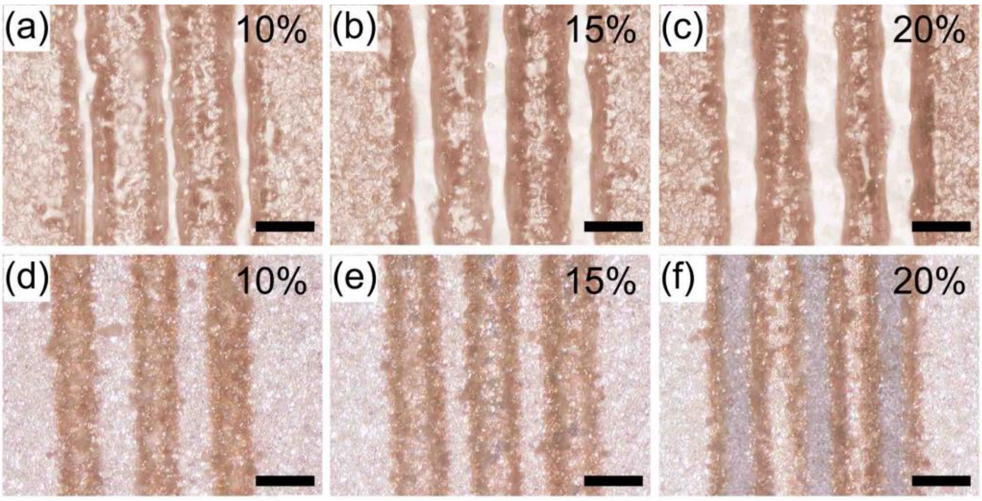

Supplementary Figure 28. (a) ~ (c) Optical images of the laser-scribed circuit lines of the FD III based ECC (50 wt% of silver) (left to right: laser power of 10%, 15%, 20%); (d) ~ (f) Optical images of the laser-scribed circuit lines of the commercial ECC (FTL-630LE, 75 wt% of silver) (left to right: laser power of 10%, 15%, 20%). (Both ECCs samples are screen printed and cured on PET, all scale bars are 20 μm.)

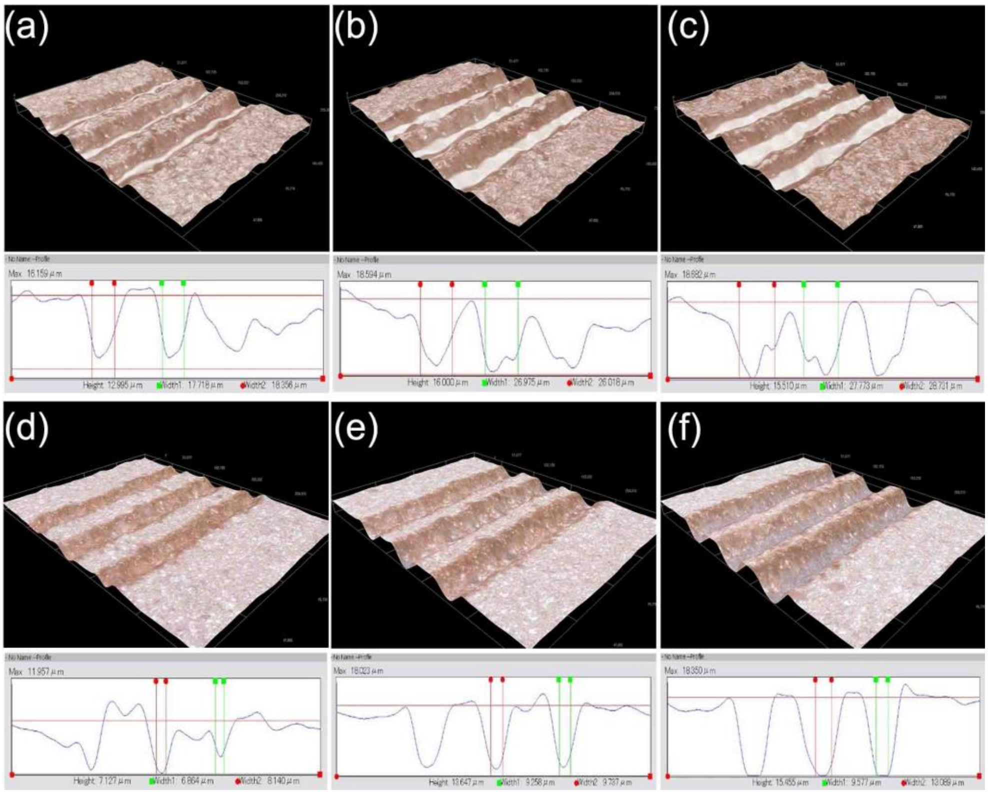

Supplementary Figure 29. Recovered 3D morphologies and the corresponding cross section height profiles. (a) ~ (c) FD III-ECC, with the laser power of 10%, 15%, 20%; (d) ~ (f) commercial ECC (FP FTL-630LE, Korea), with the laser power of 10%, 15%, 20%.

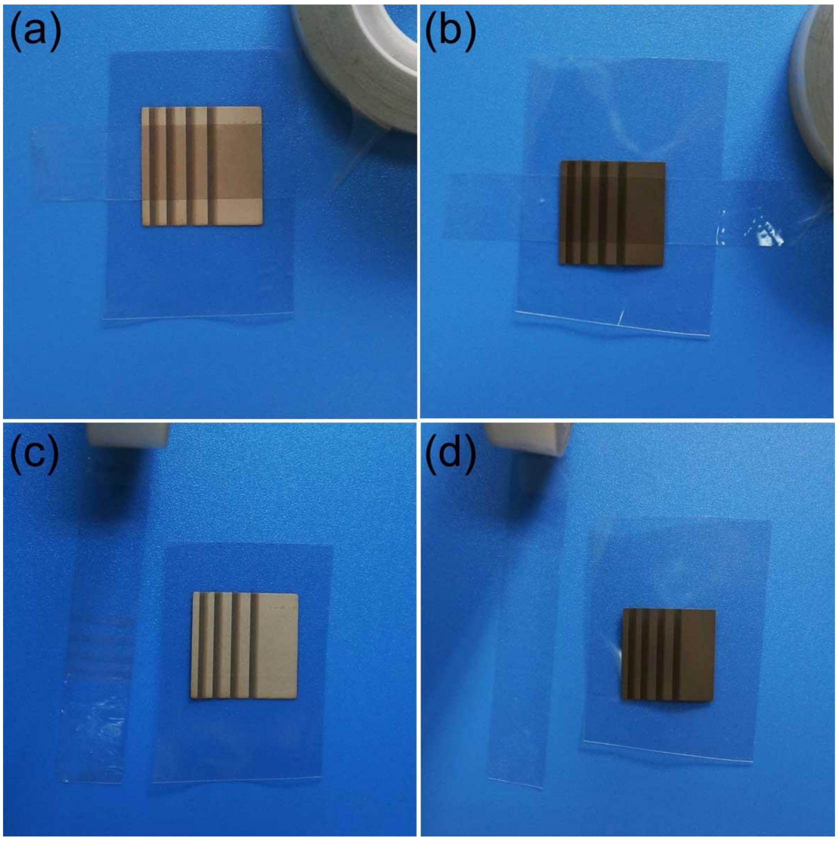

Supplementary Figure 30. Tape test of the ECC samples. (a) commercial FTL sample before tape test; (b) FD III based ECC sample before tape test; (c) commercial FTL sample after tape test (note that some parts of the same is transferred to the tape); (d) FD III based ECC sample after tape test (note that nothing is transferred to the tape visually).

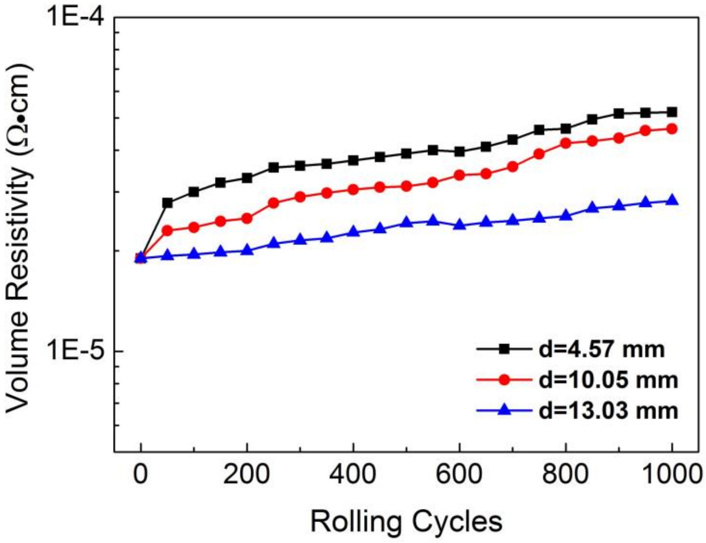

Supplementary Figure 31. Variation of volume resistivity versus bending cycles. *d* is the bending diameter.

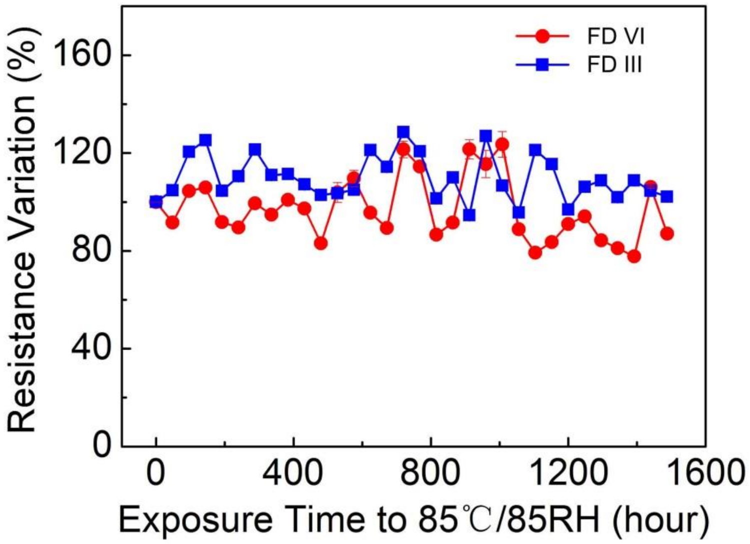

Supplementary Figure 32. 85 °C/ 85 RH relative humidity reliability test (1500 h) of the ECCs filled with FD IIII and FD VI; the silver content was 10 wt%.

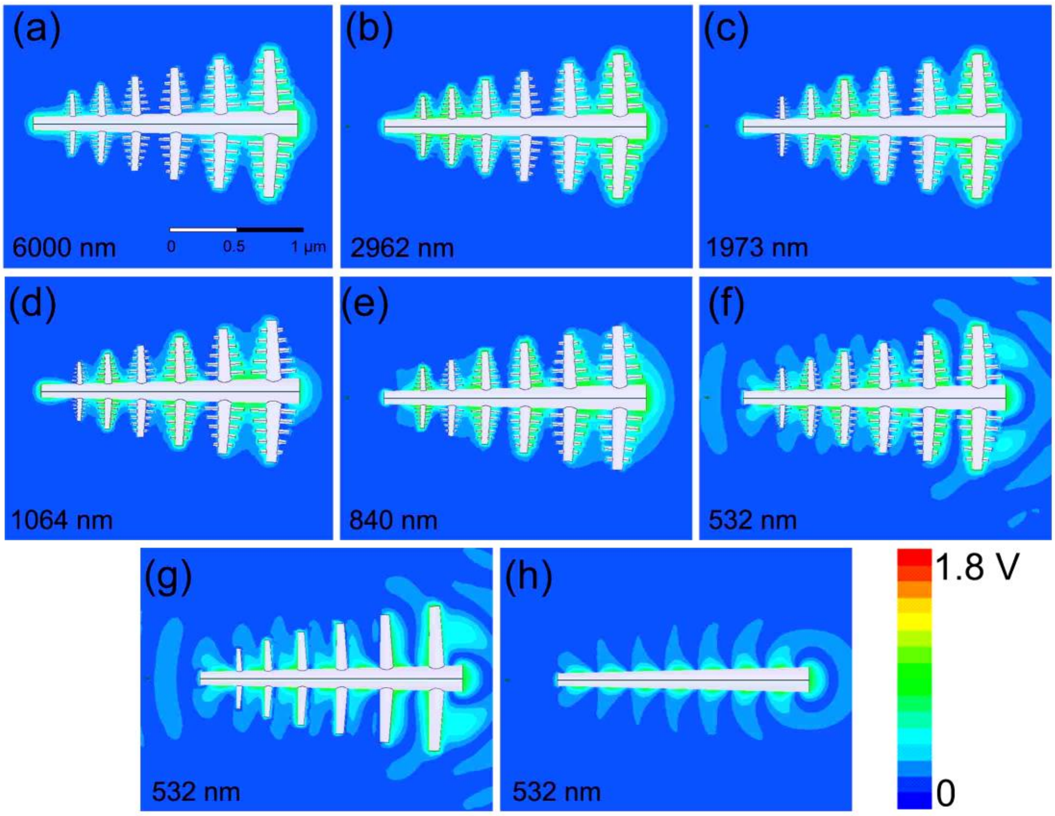

Supplementary Figure 33. Cross sectional images showing the distribution of EM field around an isolated FD primary branch. (a) ~ (f): EM field on FD VI fishbone model, with the corresponding EM wavelengths of 6000 nm, 2962 nm, 1973 nm, 1064 nm, 840 nm, and 532 nm. (g) EM field on FD III fish-bone model (EM wavelength 532 nm), (h) EM field on FD I bare rod model (EM wavelength 532 nm).

Supplementary Tables

Supplementary Table 1. Size distribution and morphology characters of various FDs.

FD types	Size distribution (μm)		Number of primary branches
	≥ 50%	≥ 90%	
FD I	1.5-2.5	1.2-2.7	8-12
FD II	1.8-3.0	1.6-3.0	8-12
FD III	4.0-5.2	3.2-5.6	12
FD IV	3.5-6.5	4.5-5.5	14-18
FD V	5.0-6.8	3.8-7.4	12-14
FD VI	6.0-8.0	5.0-8.0	18-24

Supplementary Table 2. The calculated results of the ratio of change of the interlayer distance (Δd_{12}, Δd_{23}) and surface energy for Ag (111), Ag (110) and Ag (100). Other previous theoretical and experimental results are given for comparison.

**Crystal Surface**		**Δ d_{12} (%)**	**Δ d_{23} (%)**	**γ (J m^{-2})**
Ag (111)	This work	-0.3	-0.8	0.58
	Ref.[9]	-0.2	-0.6	/
	Ref.[6]	-0.51	-0.55	0.62
	Expt.[12, 13, 14]	<|2|	0.6	0.55
Ag (110)	This work	-7.2	3.9	1.21
	Ref.[6]	-7.7	2.8	/
	Ref.[6]	-10.21	4.81	1.22
	Expt.[14, 15]	−7.8 ± 2.5	4.3 ± 2.5	1.18
Ag (100)	This work	-1.5	0.7	0.82
	Ref.[9]	-1.6	0.9	/
	Ref.[6]	-1.89	0.55	0.80
	Expt.[14, 16]	0.0 ± 1.5	0.0 ± 1.5	0.88

Supplementary Table 3. Adsorption energy (E_{ad}) and N-Ag distance ($d_{N\text{-}Ag}$) of NH_2OH adsorption on (4 × 4) silver surfaces. The N-Ag distance is defined as the distance between the N atom in NH_2OH and its underlying Ag atom.

**Adsorption site**		E_{ad} **(eV)**	$d_{N\text{-}Ag}$ **(Å)**
Ag (111)	T	0.27	2.44
	B	0.16	2.91
	fcc	0.13	3.05
	hcp	0.14	3.03
Ag (110)	T	0.47	2.40
	SB	0.27	2.93
	LB	0.12	2.91
	H	0.10	3.35
Ag (100)	T	0.29	2.42
	B	0.19	2.86
	H	0.15	3.08

Supplementary Table 4. Coverage, adsorption energy (E_{ad}) and N-Ag distance ($d_{N\text{-}Ag}$) for NH_2OH adsorbed on top sites of different Ag crystal facets with varied coverage of molecules.

	**Slab**	**Coverage**	E_{ad} **(eV)**	$d_{N\text{-}Ag}$ **(Å)**
Ag (111)	2 × 2	0.25	0.34	2.43
	3 × 3	0.11	0.34	2.44
	4 × 4	0.06	0.27	2.44
Ag (110)	2 × 2	0.25	0.51	2.40
	3 × 3	0.11	0.51	2.39
	4 × 4	0.06	0.47	2.40
Ag (100)	2 × 2	0.25	0.41	2.43
	3 × 3	0.11	0.40	2.41
	4 × 4	0.06	0.29	2.42

Supplementary Table 5. A comparison of the recently reported electrical conductive composites.

Fillers	Filler loading (wt%)	Electrical resistivity (Ω•cm)	Curing parameters		Additional condition	Reference
			Temperature (°C)	Time (min)		
Ag FD III	70	1.08×10^{-5}	150	20	Iodine treatment	Current work
	40	5.08×10^{-5}				
	8	4.9×10^{-3}				
Ag FD VI	70	1.40×10^{-5}	150	20	Iodine treatment	Current work
	40	7.80×10^{-5}				

	10	3.6×10^{-3}				
Ag FD I	70	2.73×10^{-5}	150	20	Iodine treatment	Current work
	20	9.7×10^{-3}				
Ag NPs	56	3.65×10^{-1}	RT	10	UV-cured resin	Wu et al.[20]
	75	7.5×10^{-4}				
Ag NWs	56	1.2×10^{-4}				
Ag NWs	16.9	4.0×10^{-4}	130 °C for 1 h and then 160 °C for 2 h		Mixed with SiO_2 NPs	Nam et al.[21]
Ag Fs	80	2.5×10^{-5}	150	30	Silver carboxylate coated Ag flakes and reduction by diglycidyl ether of polypropylene glycol	Zhang et al.[22]
Ag Fs	85	8.0×10^{-5}	50	15	$NaBH_4$ treatment	Yang et al.[23]
	75	3.5×10^{-4}				
Ag Fs	75	5.9×10^{-6}	150	30	Iodine treatment	Yang et al.[24, 25]
	27.5	4.8×10^{-4}				
Ag Fs	20	4.6×10^{-4}	120	60	Self-synthesized	Wang et al.[26]
Ag NPs + Ag Fs	80	2.44×10^{-2}	150	60	Decomposition of silver oxide; sintering	Zhang et al.[27]
		4.8×10^{-5}	180			
Ag NPs + Ag Fs	80	5.0×10^{-6}	150	90	Carboxylic acid treatment of Ag NPs	Jiang et al.[28]
Ag NWs + Ag Fs	75	3.3×10^{-5}	200	30	Dicarboxylic acid modification; sintering	Zhang et al.[29]
		3.2×10^{-5}	250			
		5.8×10^{-6}	300			
Ag coated Cu Fs	80	2.4×10^{-4}	150	60	Modified by an amine-based silane coupling agent	Zhang et al.[30]
Cu Fs	80	1.28×10^{-3}	130	30	Silane coupling agent	Yim et al.[31]
Cu Fs + Cu NPs	80	7.5×10^{-4}				
SWCNT	20	1.7×10^{-2}	—	—	—	Sekitani et al.[32]
SWCNT	15.8	4.8×10^{-4}	—	—	—	Sekitani et al.[33]
nAg-SWCNT + Ag Fs	38.1	9.8×10^{-3}	—	—	—	Chun et al.[34]

Note: the bulk density of carbon nanotube is about 1.3 g cm^{-3},[35] while the bulk density of silver is 10.49 g cm^{-3}.

Supplementary Table 6. Parameters of f_c, k and n of various materials in equation (4).

Material	f_c (vol%)	*Relative silver weight content at f_c (wt%)*	n	k
Ag flake	3.43	24	-1.22	3.07×10^{-6}
FD I	2.73	20	-1.47	1.79×10^{-6}
FD III	0.97	8	-1.30	1.41×10^{-6}
FD VI	1.03	8.5	-1.21	2.97×10^{-6}

Supplementary Table 7. The size and feature dimensions used in simulation model, and the dimensions are taken from the mean value on experimental measurements of the corresponding units.

Model units	Type modeled	Size (μm)	Primary branches		Secondary branches		Remarks
			Count	Width (nm)	Count	Length (nm)	
(a)	FD I	2.0	12	66	-	-	-
(b)&(c)	FD III	4.4	12	49	5-11	150-550	Various instances of secondary features
(d)	FD VI	7.0	24	78	5	450	No tertiary features
(e)	Ag crystal	2.0	-		-	-	-
(f)	Ag flake	4.0	-		-	-	Thickness 200 nm

Supplementary Discussion

Influence of temperature on preparation of FDs. As shown in Supplementary Fig. 3, we selected FD III and FD VI based ECCs with silver content of 20 wt% for the resistivity measurement at heating rate of 5°C min^{-1}. The results showed that the volume resistivity of two types of FDs based ECCs decreased firstly with the rising temperature, and then increased abruptly at higher temperature. When the temperature reached to about 150°C, the resistivity of FDs based ECCs decreased obviously. While the resistivity of FDs based ECCs kept slightly variation at the temperature range from 150°C to 250°C. These results indicated that the FDs conductive networks could maintain stable at 150°C ~ 250°C. Moreover, the FD VI based ECCs obtained the lowest volume resistivity at about 200°C, which was slightly higher than

that of FD III based ECCs. These results indicated that the FD III-ECCs were prone to form better ohmic conductive network at lower temperature as compared with the FD VI-ECCs. Moreover, the FD VI-ECCs degraded faster than the FD III-ECCs when further raising the temperature, which indicated less thermal stability for the higher fractal dendrite structures for high temperature applications.

Structural models of DFT calculation. In order to investigate the adsorptive behavior of NH_2OH molecules on silver surface, periodic slab model with the vacuum layer of 15 Å is adopted, including 2 × 2, 3 × 3 and 4 × 4 unit cells for varying coverage of NH_2OH on Ag (111), Ag (110) and Ag (100) surfaces. All these periodic supercells include seven layers of Ag atoms, of which the bottom three layers are fixed to the calculated bulk crystal structure. The remaining four top surface layers and the NH_2OH molecules are allowed to fully relax. Those highly symmetric sites on each surface are considered for adsorption of NH_2OH, i.e., the top (T), the bridge (B), the *fcc* hollow (*fcc*) and the *hcp* hollow (*hcp*) sites for Ag (111) surface; the top (T), the short-bridge (SB), the long-bridge (LB) and hollow (H) sites for Ag (110) surface; and the top (T), the bridge (B) and hollow (H) sites for Ag (100) surface, as shown in Supplementary Fig. 5.

DFT Computational details. All calculations were performed within the framework of density functional theory (DFT) as implemented in the Vienna ab initio Simulation Package (VASP).[1] The projected augmented wave (PAW) method was used to describe the interaction between valence electrons and ion cores.[2, 3] The exchange-correlation potentials were treated by the generalized gradient approximation with Perdew-Burke-Ernzerhof functional.[4] The energy cutoff for the plane-wave basis set was 500 eV. The Brillion zone was sampled 7 × 7 × 1 grid meshes for the 2 × 2 slab, 5 × 5 × 1 grid meshes for the 3 × 3 slab, and 3 × 3 × 1 grid meshes for the 4 × 4 slab by using Γ-centered scheme during the calculation. The Hellmann-Feynman force acting on each atom was converged to below 0.01 eV $Å^{-1}$ for geometrical optimization.

To check the reliability of the above methods, several tests were performed. The optimized geometry of the isolated

NH_2OH molecule is shown in Supplementary Fig. 6a. The structural parameters are as follows: *d*(N-O) = 1.459 Å, *d*(O-H) = 0.974 Å, *d*(N-H) = 1.026 Å, θ(H-O-N) = 101.9°, θ(H-N-O) = 103.4° and θ(H-N-H) = 105.2°. The above results are in agreement with experimentally reported values of 1.453 Å, 0.962 Å, 1.020 Å, 101.4°, 103.3° and 107.0°, respectively.[5] The highest occupied molecular orbital (HOMO) and lowest unoccupied molecular orbital (LUMO) are shown in Supplementary Fig. 6b and c, respectively. The distribution of frontier molecular orbital of NH_2OH shows that a covalent bond will form between the N atom and Ag atom for the NH_2OH adsorption on the surface of silver, a common trend of many open-shell atoms and small molecules adsorption on the metal surface.[6, 7, 8, 9, 10]

For the lattice constant of bulk silver, the calculated value is 4.15 Å, which is in agreement with the experimental value 4.0855 Å with an error of 1.54%. The calculated cohesive energy of silver is 2.49 eV, which is consistent with Stachiotti's result (2.52 eV).[9] We also investigated the structural properties of the clean Ag (111), Ag (110) and Ag (100) surfaces and the results are listed in Supplementary Table 2. The ratio of change of the interlayer distance is defined as:

$$\Delta d_{ij} = (d_{ij} - d_0) / d_0 \quad (1)$$

where, d_{ij} is the interval between layer *i* and *j*. d_0 is the interlayer spacing in the bulk. The negative number here means contraction of the interlayer distance as compared to the bulk structure, and vice versa. The spacing between the first and second layer decreases for all the three surfaces, but that between the second and third layer increases for (110) and (100) surfaces. It is found that the largest relaxation is on (110) surface, because it is the most loosely packed one among all three surfaces.[5] Moreover, surface energy (γ) can be calculated by:

$$\gamma = \frac{E_{slab} - 0.5NE_{Ag}^{bulk} - 0.5E_{slab}^{unrel}}{A} \quad (2)$$

where E_{slab} is the total energy of the slab, E_{Ag}^{bulk} is the energy per atom in the bulk of silver, *N* is the total number of Ag atoms contained in the slab, E_{slab}^{unrel} is the energy of the un-relaxed slab, and *A* is the surface area. Since the upper part of the slab is allowed to relax while the rest is fixed to the bulk positions, the factors 0.5 are introduced in the above equation.[11] The calculated surface energies of Ag (111), Ag (110) and Ag (100) are also listed in Supplementary Table 2,

which are consistent with other theoretical and experimental results. Moreover, the (111) surface is more stable than the other two surfaces in agreement with previous experimental and theoretical results.[6, 13-18]

Adsorptive behavior of NH_2OH molecule. The adsorption energy of NH_2OH molecule (E_{ad}) on silver can be defined as:

$$E_{ad} = E_{NH_2OH} + E_{Ag} - E_{NH_2OH/Ag} \qquad (3)$$

where, E_{NH_2OH} , E_{Ag} and $E_{NH_2OH/Ag}$ are the total energies of isolated NH_2OH molecule, Ag slab and the combined NH_2OH/Ag systems, respectively. The positive value here means that the process is exothermic. The results of E_{ad} of NH_2OH molecule adsorption on 4 × 4 silver surfaces are listed in Supplementary Table 3. It shows that, among all possible adsorption sites, the top site is the most energetically favorable one for all three surfaces. It can be seen in Supplementary Fig. 7 that the NH_2OH is tilted to the surface, which makes the binding between the NH_2OH and silver surface stronger than the one where NH_2OH is in upright or parallel to the surface. Moreover, the adsorption energies of NH_2OH molecules adsorbed on Ag (111), Ag (110) and Ag (100) surfaces are 0.27 eV, 0.47 eV and 0.29 eV, respectively. This indicates that the NH_2OH molecule prefers to be adsorbed on Ag (110) surface.

Moreover, to investigate the adsorptive behavior of NH_2OH on Ag surface, we performed the calculations by using several different slab models for the (111), (110), and (100) surfaces, respectively. The results of adsorption energies of NH_2OH at different coverages are shown in Supplementary Fig. 8 and Supplementary Table 4. It shows that the adsorption energy increases significantly with NH_2OH concentration and reaches the maximum value with a coverage rate of about 0.15. The top sites of Ag (110) are preferred adsorption at higher coverage, and the next option is Ag (100), while the last Ag (111) surfaces show repelling behavior, which represents that NH_2OH molecules tend to bind to the Ag (110) and (100) surfaces, passivating the growth on (110) and (100) surfaces of the silver nano-crystals and promoting the growth along (111) surfaces.

In our experiment, hydroxylamine plays a key role in the reactions as both the reductant and surface coupling agent.

When we reduce the concentration of hydroxylamine, the nucleation process of silver crystal decreases and the seed crystals tend to grow into more basic structures (such as FD I). By contrast, when we increase the concentration of hydroxylamine to a large extent, more complicated structures (such FD VI) are obtained. Further analysis is under exploration.

Analyses of the crystal structure and surface state of FDs. The peak positions of the three different samples (FD III, FD VI and commercial Ag flake) in the XRD spectra are similar and the observed diffraction peaks originated from (111), (200), (220), (311) and (222) diffractions (Supplementary Fig. 9a), which correspond to the typical peaks of face-centered cubic (*fcc*) structure of silver. The intensity ratio of (111) to (200) of samples FD VI, FD III and Ag flake are 5.11, 4.74, and 3.13 respectively (JCPDS: 2.1). It suggests that the preferred orientation of all three samples is the (111) lattice and FD VI has the highest preference of (111). XPS sprectra (Supplementary Fig. 9b) of the FDs correspond to the Ag 3d spectrum region. Two peaks at binding energies of 374.5 eV and 368.5 eV were observed, corresponding to the Ag $3d_{3/2}$ and Ag $3d_{5/2}$ spectrum regions, and the splitting of the 3d doublet is 6.0 eV, which is typical for metallic Ag.[17]

TEM analyses of morphologies of FDs. The TEM images of FD III and FD VI are shown in Supplementary Fig. 10, from which we can observe that the size of the secondary branches is about 500 nm and the nano-sized rim of FD VI is around 50 nm.

Characterization of FDs as the SERS Substrates. FDs have abundant nano-tip structures, some of which may act as “hot spots” for the surface enhanced Raman scattering (SERS) enhencement. Supplementary Fig. 11 displays the SERS spectra of Rhodamine 6G (R6G) on the surface-modified silver samples (FD III, FD VI, and Ag flake). From this spectra,

we can observe that the Raman spectra of the 10^{-6} M R6G adsorpted silver samples were dominated by the relatively strong peaks at 1648, 1566, 1508, 1361, 1280, 1197, 1077, 936, 767, 622 and 421 cm^{-1}, which agreed well with the previous report.[19] The intensity of the peaks of FD III and FD VI were much higher than that of the Ag flake. Thus, FDs might have potential application in SERS enhancement.

BET analysis of FDs. In order to better investigate the characteristic of specific surface area (SSA) of the FDs, we analyzed the samples by Brunauer-Emmett-Teller (BET). Supplementary Fig. 12 shows the nitrogen adsorption isotherm of the as-prepared FDs; the featured hysteresis of the isotherm between desorption and adsorption branches indicates the presence of mesopores.[18] The SSA of FD III and FD VI were 4.6128 m^2 g^{-1} and 6.3122 m^2 g^{-1}, respectively. By contrast, the SSA of Ag flake was too small to be measured (not shown here). The results can well confirm that these micro-sized FDs have a relatively large specific surface area than the commercial Ag flakes.

Evidence of sintering of adjacent FDs. The morphology of FD III after sintering at 150 °C is shown in Supplementary Fig. 14. The adjacent nano-sized rims at the outmost FD III structure were fused with each other with the neck diameter about 100 nm, while the whole FD III can maintain the initial dendritic 3-D morphology. The main part of the FDs was stable enough to endure the temperature of 150 °C.

Detailed SEM analysis of the sintering process of some powder samples. Considering the fact that there are plenty of the outmost nano-structures, we evaluated the morphology of FDs after annealing at different temperatures (as shown in Supplementary Fig. 15 and 16). FD VI was annealed in argon at 40 °C, 60 °C, 80 °C, 100 °C, 120 °C, 140 °C, 160 °C, 180 °C, 200 °C, 220 °C, 250 °C and 300 °C for 30 min, respectively for detailed observations. The annealing temperature of both FD III and Ag flake were set to 60 °C, 100 °C, 150 °C, 200 °C, 250 °C and 300 °C for 30 min, respectively.

As for FD III, the secondary structure of every branch was almost kept intact after annealing below 100 °C, which is different with the sintering temperature of tertiary fractal structure of FD VI (the lowest sintering temperature is about 60 °C). With the temperature rise to over 100 °C, the silver grains on the secondary structure began to transfer to the primary structure, clearly indicating the sintering process (as shown in Supplementary Fig. 15b). When the temperature rise to 150 °C, the primary structure became thicker, with the decreased surface roughness and the disappearance of the nano-structure (see Supplementary Fig. 15c). Furthermore, the primary branches changed from dendritic ones to micro-particles at 250 °C (see Supplementary Fig. 15e) and the nanostructures on tips of adjacent silver particles seriously sintered with each other.

The SEM images of the control Ag flakes after different annealing conditions are shown in Supplementary Fig. 17. The morphology and aggregation of Ag flakes barely changed below 200 °C. When the temperature rise to 200 °C and above, the surface of Ag flakes turned smooth and the edges of the adjacent flakes sintered together at about 250 °C, which is considerably higher than that of FDs.

TGA/DSC analysis. To study the thermal stability behavior of the FDs, thermal gravimetric analysis (TGA) and differential scanning calorimatry (DSC) were carried out in argon atmosphere with the gas flow rate of 2 °C min^{-1} and the ramping temperature from 25 °C to 300 °C (Supplementary Fig. 18). The weight losses for both FD III and FD VI were less than 0.6 % at 300 °C, which suggested that the surface of FDs was clean. DSC analysis showed accelerated endothermic behavior in the temperature range (Supplementary Fig. 18).

Structural integrity of FDs in dispensing process. In order to investigate the feasibility in practical dispensing process, the as-prepared FDs, e.g. FD III was sonicated in a bath (the power was 100 W) for 30 min in ethanol. From the SEM analysis, the fractal structure of the FDs was maintained very well (Supplementary Fig. 19), even though some of the edge of the branches were slightly blunted due to the onset of minor thermal energy. The robustness of the FDs ensures

them to be mixed with a resin binder in an optimum processing condition. (Roll milling is not recommended.)

Cross sectional SEM images of FD based ECCs. The cross sectional SEM images of the FD based ECC samples with different Ag contents are shown in Supplementary Fig. 20 and 21. The distribution of the FDs in the polymer matrix is clearly exhibited. The relatively uniform distribution guaranteed the stability and isotropicity of the electrical conduction of the ECCs. By contrast, commercial Ag flakes dispersed in the resin matrix are shown in Supplementary Fig. 22. To be noted, Ag flakes often tend to orient along the shearing direction which ends up with a certain level of anisotropicity in conventional binder mixing processes.

A comparison of recently reported electrically conductive composites. The resistivity of the recently reported ECCs data are summarized in Supplementary Table 5 for a more detailed comparison, including the ECCs based on Ag NPs, Ag NWs, Ag flakes, Cu NPs, Cu flakes and carbon materials etc. The FD based ECCs in this work show ultralow percolation threshold with relatively low electrical resistivity, as compared with the other ever-reported data. Among these reports, the studies on SWCNT and mixture of SWCNT and silver nanowires are listed as the references.

Calculation of the simulated percolation curve. Percolation theory was used to generate the simulated curve in Fig. 5a, using equation (4) ~ (6)

$$\rho = k(f - f_c)^n \qquad (4)$$

where ρ is the resistivity, k is a linear proportionality constant, f is the volume fraction, f_c is the critical volume fraction at percolation, and n is a fitting exponent. The critical volume fraction f_c is influenced by particle size, shape and morphology.

The calculation of volume fraction was according to equation (5) and (6):

$$f = \frac{V_{Ag}}{V_{Epoxy} + V_{Ag}} \qquad (5)$$

$$\frac{V_{Ag}}{V_{Epoxy}} = \frac{M_{Ag}}{M_{Epoxy}} \times \frac{\rho_{Epoxy}}{\rho_{Ag}} \qquad (6)$$

where V_{Ag} and V_{Epoxy} are the volumes of the Ag fillers and epoxy resin respectively; M_{Ag} and M_{Epoxy} are the mass contents, and ρ_{Ag} and ρ_{Epoxy} are the densities of bulk silver (10.49 g cm^{-3}) and epoxy resin (1.18 g cm^{-3}). Considering that the presence of nano-structure in the FD fillers reduces the macroscopic density of the metal, a slightly over-estimation of the volume of Ag fillers may occur here.

A least square regression fit on the measured data was performed to find k (linear proportionality constant) and n (fitting exponent). The least square fit was performed by first linearizing the equation (4) to:

$$\log \rho = \log k + n \cdot \log (f - f_c) \qquad (7)$$

Then we can determine k and n from the y-intercept and slope of the fitted curve respectively. As for FD I, the best-fit curve was obtained by taking f_c = 0.0273 (corresponding to the Ag weight content of 20 wt%) as it is where the ECC shows the most abrupt change. Using this method, we found that k = 1.79×10^{-6} and n = -1.47. The detailed parameters of this percolation equation are shown in Supplementary Table 6 and the least squares linear fits are shown in Supplementary Fig. 23.

Structural models and Computational methods of 3-D Monte Carlo percolation simulation. A 3-D Monte Carlo simulation using the classical Hoshen-Kopelmen algorithm was employed to study the percolation probability of the ECC samples.[36] In order to ensure our models more close to the real situation, we constructed the models according to the SEM observations. FD I, FD III, and FD VI were modeled and scattered into the simulation domain randomly for observation of the percolation phenomena; Ag spherical particles and Ag flakes are separately simulated as the control samples. Thousands of the modeled particle units with 3 degrees of rotational freedom, each of which consists of millions of nodes, were randomly positioned into a 3-D simulation domain.

The crystal models used in the 3-D Monte Carlo computation is shown in Supplementary Fig. 24, including FD I (with primary feature), FD III (with primary and secondary fractal features), and FD VI (with primary and secondary fractal features; tertiary fractual features were reasonably neglected for simplification) models. Ag spherical particles and Ag flakes are also modeled as the control. In order to minimize the effect of geometrical discrepancies of the modeled FDs and the real FD I, FD III, and FD VI particles; the modeling parameters and average values (Supplementary Table 7) are carefully chosen so as to statistically approximate to the real particles. Surface effects, such as iodine treatment effect and other contact resistances,[24] are ignored in the simulation to simplify the computation, which lead to minor underestimation of the percolation probability.

The particle models used in the simulation are constructed in a L^3 space, where L is the typical size of the concerned particles. When considering from the smallest dendritic features (about 20 nm) to the largest macroscopic composite thickness (being set to about 100 μm), a high resolution simulation (Fig. 5d) is necessary to cover all the huge range of length scales in the particle system. In this modeled simulation, typical results are obtained from a $11L \times 5L \times 5L$ to $20L \times 10L \times 10L$ (depends on particle type) periodic domain with the total number of nodes reaching one billion, and the results attain a sufficiently high resolution to resolve the nano-scale features while maintaining the macroscopic statistical properties of the particle system. The domain size is chosen thru a domain size test, by observing the stability of statistic at each mass fraction points at increasing domain size. It is observed that the percolation statistic converges when domain size reached the range $10L$-$20L$. This is consistent with some classical numerical studies on percolation.[37] In general, more complex shape requires larger domain and more systems for each mass fraction point to stabilize the percolation statistic. The total number of system simulated for a single particle shape is about 4000 to 12000 systems. Each single system has about 0.1 to 1.0 billion nodes. For each shape, we compute a number of systems (100 to 400) at each mass fraction point to provide stable statistics, and only limited statistical fluctuation is observed in the percolation probability curves. If the curves are not sufficiently smooth with respect to the percolation threshold range obtained, we increase the

number of system calculated until a stable statistic is obtained at each mass fraction point.

Computational results of percolation. The overall silver mass content of each dendrite crystal in the L^3 unit is set to about 6.7 ~ 15.4 wt% (taking the filler density as the one for bulk silver, i.e. 10.49 g cm^{-3}). When the above models are used for the Monte Carlo simulation, the percolation state curves are drawn versus silver mass fractions (Fig. 4c). The resulting curves show that all FDs, regardless of the secondary features, critically percolate at around 12.8 ~ 18.5 wt%, while those of the spherical model (64.6 wt%) and planar flake model (47.5 ~ 66.0 wt%) require much more silver content to percolate through the domain. As for the simulated results, the percolation probability of the spherical model appeared to be close to the theoretical criticality value (i.e. the Scher-Zallen invariance).[38] For that of the Ag flake model, the issue of rotational freedom of Ag flakes was considered. When Ag flakes are allowed to orient randomly along the radial axis, the percolation threshold decreased (47.5 wt%) accordingly; when the Ag flakes are not allowed to rotate in that manner, the percolation threshold increased (66.0 wt%), which is consistent with previous report.[39] As shown in Fig. 4c, the pink zone marks the region between the two extremities, and the other examined flake cases with medium rotational freedom fell well between the two extreme values.

The interrelationships among different particles showed remarkable agreement with those relations observed in experiments (Fig. 4a), showing the same trend and order of percolation preference. This agreement between computational and experimental results provides evidence for the validity of our modeling on percolation.

Evaluation of device applications of FD based ECCs. Currently, most of the packaging methods for the touch panel sensor module for smartphones and tablet PCs include G + F (glass front cover plus film module), G + G (glass front cover plus glass module), OGS (one glass solution), On-Cell (on color filter) and In-Cell (in TFT-LCD module) etc. Among them, thin film based modules have become the mainstream (about 48% and 94% of the global market shares for

smartphone and tablet PC in 2014, respectively).[40] In order to realize finer border and better film transparency, polymer films e.g. PET are often used as the common substrate for the film module (as shown in Fig. 5b). In order to evaluate the feasibility of the FD based ECC samples in touch panel applications, we adopted a benchmark commercial silver paste (FP FTL-630LE, Korea, polyester resin based) as the control sample and systematically evaluated the wiring performance of the FD based ECC samples for such application.

In order to better compare the samples, we adopted polyester resin for the FD based ECCs, which showed equal electrical conductivity to the epoxy based FD-ECC. FD III-ECC (50 wt% of silver) was screen-printed on PET substrate (shown as Supplementary Fig. 25) with the same thickness as the commercial silver paste (FTL-630LE, FP, Korea, 75 wt% of silver). Both samples were cured at 150 °C for 20 min, and then were laser-scribed with different power (StrongLaser Co., laser wavelength: 1064 nm, straight line processing speed: 1000 mm s^{-1}, maximum laser power 20 W, a photographic image of the laser micro-processing system is shown in Supplementary Fig. 26). In order to ensure adequate wiring capability (20 μm width fine lines up to decimeter length scale), 30% of the laser power is required for the benchmark ECC; while for FD III-ECC, only 15% of the laser power is enough. Supplementary Fig. 28 and 29 showed the optical microscopic images of the laser-scribed ECCs (KH-7700 3-D video microscope). FD III-ECC sample had regular patterns with the laser power of 15%, which showed similar patterning resolution to that of the commercial one patterned with 30% (not shown here). The volume resistivity of the laser-scribed circuit line is summarized in Supplementary Fig. 27, suggesting that FD III-ECC possesses excellent conductivity, especially at low laser power levels. The above results indicated that the FD-ECCs have excellent potential in the flexible display applications with low cost and high performance.

Adhesion test of FD based ECCs. The tape test was conducted to testing the adhesion of the FD III based ECCs with the PET substrate, as shown in Supplementary Fig. 30. We chose the laser-scribed FD based ECC sample, and the FTL silver paste as control sample. As for the FD based ECC sample, the conductive pattern remains intact and unnoticeable

ECC material could be removed by the tape, while the apparently FTL ECC sample can be scraped off, as shown on the tape. This can be ascribed to the excellent mechanical strength of the FD based ECCs and the excellent bonding towards the PET substrate.

Bending test of FD-ECCs. In order to investigate both the mechanical robustness and electric conductivity of the FD based ECCs samples under repeatedly bending conditions, we rolled the printed FD III based ECC samples with the silver content of 50 wt% at different curvature diameters for 1000 times, and the curves of volume resistivity change versus bending cycles are shown in Supplementary Fig. 31. The resistivity increased with bending cycles, and the resistance further increased at smaller bending diameter, due to more severe deformation. The volume resistivity of the samples still maintained at 1.9 ~ 5.2×10^{-5} Ω•cm after 1000 cycles at the smallest bending diameter, i.e. 4.57 mm of bending diameter.

Thermal-humidity Reliability Test of FD-ECCs. ECC samples were evaluated by the temperature-humidity test (THT). The FD based ECCs with Ag content of 10 wt% were aged in an ESPEC SETH-Z-042L humidity chamber for 1500 h. The electrical resistances of the samples were measured by a four-point probe tester per 48 h during the test, which exhibited negligible variations (Supplementary Fig. 32). The reliability test proved that the FDs based ECCs (with as low as 10 wt% filler loading) had sufficient reliability for practical applications.

Simulation of the electromagnetic enhancement effect on a single FD branch. When illuminated by the laser beam, there are mainly two parts of electromagnetic (EM) thermal contribution for heating up the FDs. One is from the increased illuminated surface area per particle which promotes the absorption of heat accumulated from the illuminated area; another part is from the enhanced resonation with EM waves due to the presence of the unique nano-size features. Based on the finite element analysis (FEA) method, we studied the resonance situation of the EM waves on the FD nano-features. The Maxwell equations were solved by the HFSS packages from ANSYS and the Ag complex dielectric

constant was obtained from Johnson et al.[41] Based on the experimental observations summarized in Supplementary Table 1, we built the typical fractal fishbone-like models which were morphologically similar to the primary branches of various FDs, and assumed the lengths of all models were 2200 nm. The accumulation of EM field near some of the nano-structures with appropriate size was observed and compared with that of the particles having less complex features, as shown in Supplementary Fig. 33 (a) ~ (h). Based on the simulation results, under a large range of EM frequencies (from 532 nm to 6000 nm), most parts of the FD have EM resonance; particularly, thermal effect is significantly enhanced in the nano-sized regions.

Supplementary Methods

Multi-channel micro-droplet reaction system. In order to effectively manipulate the morphology and size of the precipitates and to ensure reproducibility in mass preparation, we set up a multi-channel micro-droplet reaction system, which can conveniently control the molar ratio of the reagents and the reacting rate in a continuous preparation condition. The digital photo of the platform is shown in Supplementary Fig. 1a. Typically, 2.0 L of $AgNO_3$ (0.06 M) and NH_2OH (Alfa Aesar) (0.24 M) aqueous solutions were prepared in two beakers, respectively. The solutions were pumped at the same velocity and joined together at the end of two or more needles. After forming the single droplets, the mixed liquid dropped down and continued reacting in the conical flasks array. During the reaction, the conical flasks were shaken gently at room temperature on the orbital shaker. A large amount of precipitate was observed in the conical flasks within several minutes. It was then filtered and washed by de-ionized water for three times, and then dried in a vacuum desiccator at room temperature. The as-synthesized FDs with various morphologies were shown in Supplementary Fig. 2.

Preparation of FDs. The size distribution of various FDs prepared at various conditions is shown in Supplementary Table 1. Within a broad processing window, formation of FDs with various kinds of geometric characteristic and size can

be obtained. For instance, when we controlled the molar ratio of NH_2OH to $AgNO_3$ to 1 : 1 and the pumping rate to 0.5-5 mL min^{-1}, only the urchin-like FDs with rod-like branches were obtained, named with FD I. When these two key parameters were increased to 4 : 1 and 2.5 mL min^{-1}, we obtained FD II with rod-like branches and nano-sized secondary rims grown on them. The detailed reaction conditions of these six types of FDs are shown in Fig. 1a and Supplementary Fig. 2. To be specific, FD I and FD II possess 8-12 branches, six of which are in the same plane with the angle of 60° between each other; the other branches are distributed in mirror symmetry. FD III and FD IV have 12-24 primary branches and with sencondary structures (size: 150-550 nm) on them. FD V and FD VI have 12-24 branches, the size of sencondary structures are 450 nm in average and the tertiary structures are with the size around 50 nm. The detailed size distribution of various FDs is shown in Supplementary Table 1.

Preparation of Au FDs. Besides preparing the Ag FDs, this multi-channel micro-droplet reaction system can be used to prepare Au FDs as well. For example, by controlling the molar ratio of NH_2OH to $HAuCl_6$ in the range of 2 to 40 and the pH value in the range of 4.5 to 8.0, Au FDs can be obtained in a scalable way (Supplementary Fig. 4). The as-prepared Au FDs were characterized by its primary structures sized 1 to 1.5 μm and secondary structures sized 40 to 50 nm. The morphological difference between Au FDs and Ag FDs can be attributed to the different reaction kinetics.

Supplementary References

1. Kresse G., Furthmüller J. Efficiency of ab-initio total energy calculations for metals and semiconductors using a plane-wave basis set. *Comp. Mater. Sci.* **6**, 15-50 (1996).

2. Kohn W., Sham L. J. Self-consistent equations including exchange and correlation effects. *Phys. Rev.* **140**, A1133-A1138 (1965).

3. Kresse G., Joubert D. From ultrasoft pseudopotentials to the projector augmented-wave method. *Phys. Rev. B* **59**, 1758-1775 (1999).

4. Perdew J. P., Burke K., Ernzerhof M. Generalized gradient approximation made simple. *Phys. Rev. Lett.* **77**,

3865-3868 (1996).

5. Lindeke B. The non-and postenzymatic chemistry of N-oxygenated molecules. *Drug. Metab. Rev.* **13**, 71-121 (1982).

6. Fu H., Jia L., Wang W., Fan K. The first-principle study on chlorine-modified silver surfaces. *Surf. Sci.* **584**, 187-198 (2005).

7. Kokh D. B., Buenker R. J., Whitten J. L. Trends in adsorption of open-shell atoms and small molecular fragments on the Ag(111) surface. *Surf. Sci.* **600**, 5104-5113 (2006).

8. Kilin D. S., Prezhdo O. V., Xia Y. Shape-controlled synthesis of silver nanoparticles: ab initio study of preferential surface coordination with citric acid. *Chem. Phys. Lett.* **458**, 113-116 (2008).

9. Stachiotti M. First-principles study of the adsorption of NH_3 on Ag surfaces. *Phys. Rev. B* **79**, 115405-115414 (2009).

10. Oguzie E. E., Li Y., Wang S. G., Wang F. Understanding corrosion inhibition mechanisms—experimental and theoretical approach. *RSC Adv.* **1**, 866-873 (2011).

11. Calatayud M., Minot C. Effect of relaxation on structure and reactivity of anatase (100) and (001) surfaces. *Surf. Sci.* **552**, 169-179 (2004).

12. Culbertson R., Feldman L., Silverman P., Boehm H. Epitaxy of Au on Ag (111) studied by high-energy ion scattering. *Phys. Rev. Lett.* **47**, 657-660 (1981).

13. Statiris P., Lu H., Gustafsson T. Temperature dependent sign reversal of the surface contraction of Ag (111). *Phys. Rev. Lett.* **72**, 3574-3577 (1994).

14. Smith J. R., Banerjea A. Equivalent-crystal theory of oscillatory surface relaxation. *Phys. Rev. B* **37**, 10411-10414 (1988).

15. Kuk Y., Feldman L. Oscillatory relaxation of the Ag (110) surface. *Phys. Rev. B* **30**, 5811-5816 (1984).

16. Li H., Quinn J., Li Y., Tian D., Jona F., Marcus P. Multilayer relaxation of clean Ag [001]. *Phys. Rev. B* **43**, 7305-7307 (1991).

17. Hollander J. M., Jolly W. L. X-ray photoelectron spectroscopy. *Accounts Chem. Res.* **3** 193-200 (1970).

18. Spinney P. S., Howitt D. G., Collins S. D., Smith R. L. Electron beam stimulated oxidation of carbon. *Nanotechnology* **20**, 465301 (2009).

19. Liang H., Li Z., Wang W., Wu Y., Xu H. Highly surface‐roughened “flower‐like” silver nanoparticles for extremely sensitive substrates of surface-enhanced Raman scattering. *Adv. Mater.* **21**, 4614-4618 (2009).

20. Wu H., Liu J., Wu X., Ge M., Wang Y., Zhang G*., et al.* High conductivity of isotropic conductive adhesives filled with silver nanowires. *Int. J. Adhes. Adhes.* **26**, 617-621 (2006).

21. Nam S., Cho H. W., Lim S., Kim D., Kim H., Sung B. J. Enhancement of electrical and thermomechanical properties of silver nanowire composites by the introduction of nonconductive nanoparticles: Experiment and simulation. *ACS Nano* **7**, 851-856 (2012).

22. Zhang R., Moon K-s., Lin W., Agar J. C., Wong C. P. A simple, low-cost approach to prepare flexible highly conductive polymer composites by in situ reduction of silver carboxylate for flexible electronic applications. *Comp. Sci. & Tech.* **71**, 528-534 (2011).

23. Yang C., Lin W., Li Z., Zhang R., Wen H., Gao B*., et al.* Water-based isotropically conductive adhesives: towards green and low-cost flexible electronics. *Adv. Func. Mater.* **21**, 4582-4588 (2011).

24. Yang C., Xie Y. T., Yuen M. M. F., Xu B., Gao B., Xiong X*., et al.* Silver surface iodination for enhancing the conductivity of conductive composites. *Adv. Func. Mater.* **20**, 2580-2587 (2010).

25. Yang C., Wong C. P., Yuen M. M. F. Printed electrically conductive composites: conductive filler designs and surface engineering. *J. Mater. Chem. C* **1**, 4052-4069 (2013).

26. Wang J., Chen Z., Hu Y., Jiang X., Chen D., Zhang W. Flaky silver powders prepared with nanofilm transition method: application for printable electronics. *J. Mater. Chem. C* **1**, 230-233 (2012).

27. Zhang R., Moon K-s., Lin W., Wong C. P. Preparation of highly conductive polymer nanocomposites by low temperature sintering of silver nanoparticles. *J. Mater. Chem.* **20**, 2018-2023 (2010).

28. Jiang H., Moon K-s., Li Y., Wong C. P. Surface functionalized silver nanoparticles for ultrahigh conductive polymer composites. *Chem. Mater.* **18**, 2969-2973 (2006).

29. Zhang Z., Chen X., Xiao F. The sintering behavior of electrically conductive adhesives filled with surface modified silver nanowires. *J. Adhes. Sci. & Tech.* **25**, 1465-1480 (2011).

30. Zhang R., Lin W., Lawrence K., Wong C. P. Highly reliable, low cost, isotropically conductive adhesives filled with Ag-coated Cu flakes for electronic packaging applications. *Int. J. Adhes. Adhes.* **30**, 403-407 (2010).

31. Yim M. J., Li Y., Moon K-s., Wong C. P. Oxidation prevention and electrical property enhancement of copper-filled isotropically conductive adhesives. *J. Elect. Mater.* **36**, 1341-1347 (2007).

32. Sekitani T., Noguchi Y., Hata K., Fukushima T., Aida T., Someya T. A rubberlike stretchable active matrix using elastic conductors. *Science* **321**, 1468-1472 (2008).

33. Sekitani T., Nakajima H., Maeda H., Fukushima T., Aida T., Hata K*., et al.* Stretchable active-matrix organic light-emitting diode display using printable elastic conductors. *Nat. Mater.* **8**, 494-499 (2009).

34. Chun K. -Y., Oh Y., Rho J., Ahn J. -H., Kim Y. -J., Choi H. R., *et al.* Highly conductive, printable and stretchable composite films of carbon nanotubes and silver. *Nat. Nanotech.* **5**, 853-857 (2010).

35. Behabtu N., Young C. C., Tsentalovich D. E., Kleinerman O., Wang X., Ma A. W. K., *et al.* Strong, light, multifunctional fibers of carbon nanotubes with ultrahigh conductivity. *Science* **339**, 182-186 (2013).

36. Hoshen J., Kopelman R. Percolation and cluster distribution. I. Cluster multiple labeling technique and critical concentration algorithm. *Phys. Rev. B* **14**, 3438-3445 (1976).

37. Heermann D. W., Stauffer, D. Phase diagram for three-dimensional correlated site-bond percolation, *Zeitschrift fur Physik B,* **44**, 339-344 (1981).

38. Scher H., Zallen R. Critical density in percolation processes. *J. Chem. Phys.* **53**, 3759-3761 (1970).

39. Kim W., Taya M., Nguyen M. Electrical and thermal conductivities of a silver flake/thermosetting polymer matrix composite. *Mech. Mater.* **41**, 1116-1124 (2009).

40. Hsieh C. Display Search Q3'14 Touch Panel Market Analysis Report; 2014.

41. Johnson P. B., Christy R. W. Optical constants of noble metals. *Phys. Rev. B.* **6**, 4370-4379 (1972).
